# Deconstruction of Dual-Site Tankyrase Inhibitors Provides Insights into Binding Energetics and Suggests Critical Hotspots for Ligand Optimization

**DOI:** 10.1021/acs.jmedchem.4c02845

**Published:** 2025-03-26

**Authors:** Sven T. Sowa, Murat Kücükdisli, Yelena Mostinski, David A. Schaller, Carolina S. Vinagreiro, Davide Cirillo, Chiara Bosetti, Shoshy Alam Brinch, Kirsten van Laar, Anita Wegert, Ruben G. G. Leenders, Stefan Krauss, Jo Waaler, Andrea Volkamer, Lari Lehtiö, Marc Nazaré

**Affiliations:** † Faculty for Biochemistry and Molecular Medicine & Biocenter Oulu, University of Oulu, Aapistie 7, 90220 Oulu, Finland; ‡ Medicinal Chemistry, Leibniz-Forschungsinstitut für Molekulare Pharmakologie (FMP), Campus Berlin Buch, Robert-Roessle-Str. 10, 13125 Berlin, Germany; § In Silico Toxicology and Structural Bioinformatics, Institute of Physiology, Charité Universitätsmedizin Berlin, Virchowweg 6, 10117 Berlin, Germany; ∥ Oslo University Hospital, P.O. Box 4950, Nydalen, 0424 Oslo, Norway; ⊥ Hybrid Technology Hub—Centre of Excellence, Institute of Basic Medical Sciences, University of Oslo, 0317 Oslo, Norway; # Symeres Netherlands B.V., Kerkenbos 1013, 6546 BB Nijmegen, The Netherlands; ¶ Data Driven Drug Design, Faculty of Mathematics and Computer Sciences, Saarland University, 66123 Saarbrücken, Germany

## Abstract

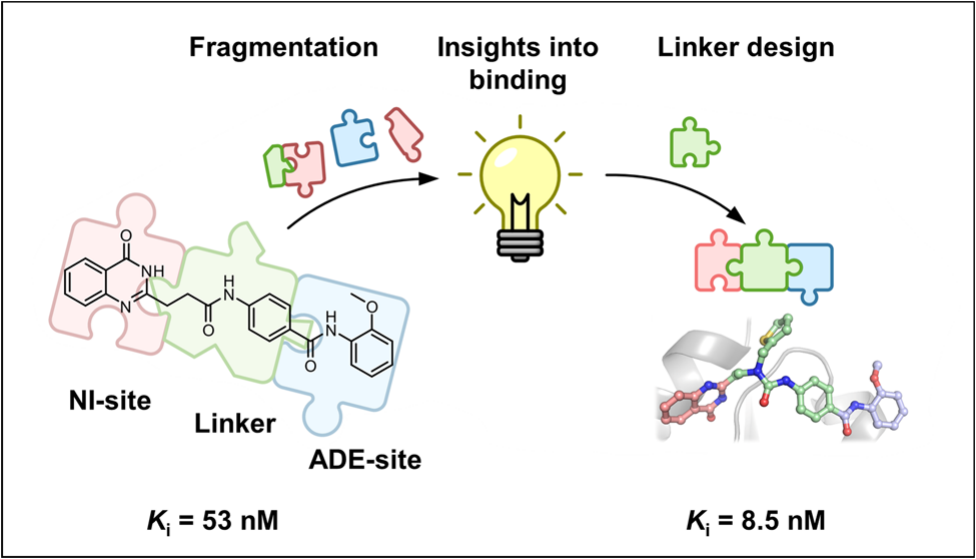

Designing inhibitors is a complex task that requires a deep understanding of protein–ligand interactions and their dynamics. Ligands often interact with multiple binding subsites, with noncovalent interactions affecting binding affinity. Conformational changes and plasticity of both, the ligand and the protein influence binding energetics. We investigated the tankyrase ADP-ribosyltransferase as a promising drug target regulating many cellular pathways. Despite the existence of diverse tankyrase inhibitors, their binding energetics and contributions of flexible cryptic subpockets to binding affinity remain elusive. To examine these aspects, we deconstructed inhibitors to key fragments, dissected their energetic contribution to the affinity, and determined their binding mode by X-ray crystallography. Varying ligand efficiencies of the deconstructed, pocket-binding fragments revealed the cryptic nature of subpockets. These insights enabled us to redesign inhibitors with novel linkers, the observed key area for optimization, increasing the potency in enzymatic and cell-based assays by 7.5-fold and 6.2-fold compared to the parent ligand.

## Introduction

There is still a considerable gap in our current understanding of noncovalent protein–ligand interactions and fragment-binding energetics, as multiple functional entities are involved in synergistic binding events. Deconvolution of composite relative energetic contributions from the observed affinity of noncovalent protein–ligand interactions remains a key challenge in accurately predicting absolute binding affinity.^1−3^ Distinguishing strong from weak interactions and resolving individual energetic contributions in complex biological systems is critical for improving the accuracy of predicting the absolute affinity of protein–ligand interactions in rational drug design. These protein–ligand interactions, however, are not monolithic and typically are noncontiguously distributed encompassing different loci (hotspots) at proximal sites, as most inhibitors address more than one (sub)site.^4^ Therefore, experimentally dissecting these different interactions within the binding sites by site-directed mutagenesis, either by point mutations or by multiple simultaneous mutations, often alters the overall conformation and influences protein stability and is therefore of limited utility.^5^ Consequently, fragment deconstruction has emerged as an important experimental strategy to analyze and map the energetic- and structural interaction patterns of a given ligand. This approach can help in determining the minimal pharmacophoric constituents and nonadditive linker contribution when fragments are joined. Moreover, such an analysis may also uncover unfavorable preorientation of key binding fragment constituents by a nonoptimal linking moiety.^6−8^

Protein dynamics, in the presence of transient, induced-fit-type cryptic pockets in regions of high flexibility while holding high functional relevance for ligand binding, adds another dimension of complexity to the overall energetic contribution, although this aspect is not completely understood.^6^ In particular, strong hotspots in proximity of a cryptic site is a common motif in protein–ligand binding architecture.^7^ In addition, quantitative studies of the influence of a linker on the total free energy of binding (Δ*G*) in the context of an induced cryptic pocket are still missing and require further investigation.

In this study, we explored the molecular features, energetic additivity and linker contributions by deconstructing two potent cognate dual-site binders addressing an induced pocket as well as an affinity pocket using a well-characterized tankyrase (TNKS) model system. This allowed us to systematically investigate the energetics by addressing a proximally induced pocket and to identify the caveats of fragment merging with a flexible linker. These findings led to the development of a series of rationally designed reconstructed inhibitors with superior affinities.

TNKSs are ADP-ribosyltransferases that catalyze the transfer of multiple ADP-ribosyl groups from NAD^+^ to their protein targets.^8^ The poly-ADP-ribosyl groups act as signals for subsequent ubiquitination, ultimately leading to the proteasomal degradation of the target proteins. The human genome encodes two TNKSs: TNKS1 and TNKS2; both these proteins have high sequence similarity and overlapping functions in the cell.^9^ TNKSs are involved in the regulation of many cellular functions such as telomere homeostasis,^10^ mitotic spindle formation,^11^ Hippo-YAP signaling,^12^ and glucose metabolism.^13^ As integral regulators of WNT/β-catenin signaling,^14^ TNKSs have received significant attention in recent years as attractive therapeutic targets for cancer and fibrosis.^8,15−17^

TNKS inhibitors are often based on molecules with a dual-binding mode to inhibit the catalytic domain, addressing two subpockets. These small molecules contain three parts accommodated in the NAD^+^-binding pocket (NI): a nicotinamide-mimicking portion, an adenosine-addressing moiety, and a linker. The structurally unique cryptic adenosine-binding pocket (ADE) of TNKS provides an excellent basis for the development of TNKS selectivity.^8,18,19^ Several TNKS antagonists with nanomolar activity and high selectivity have been reported.^20−23^

**Figure 1:**
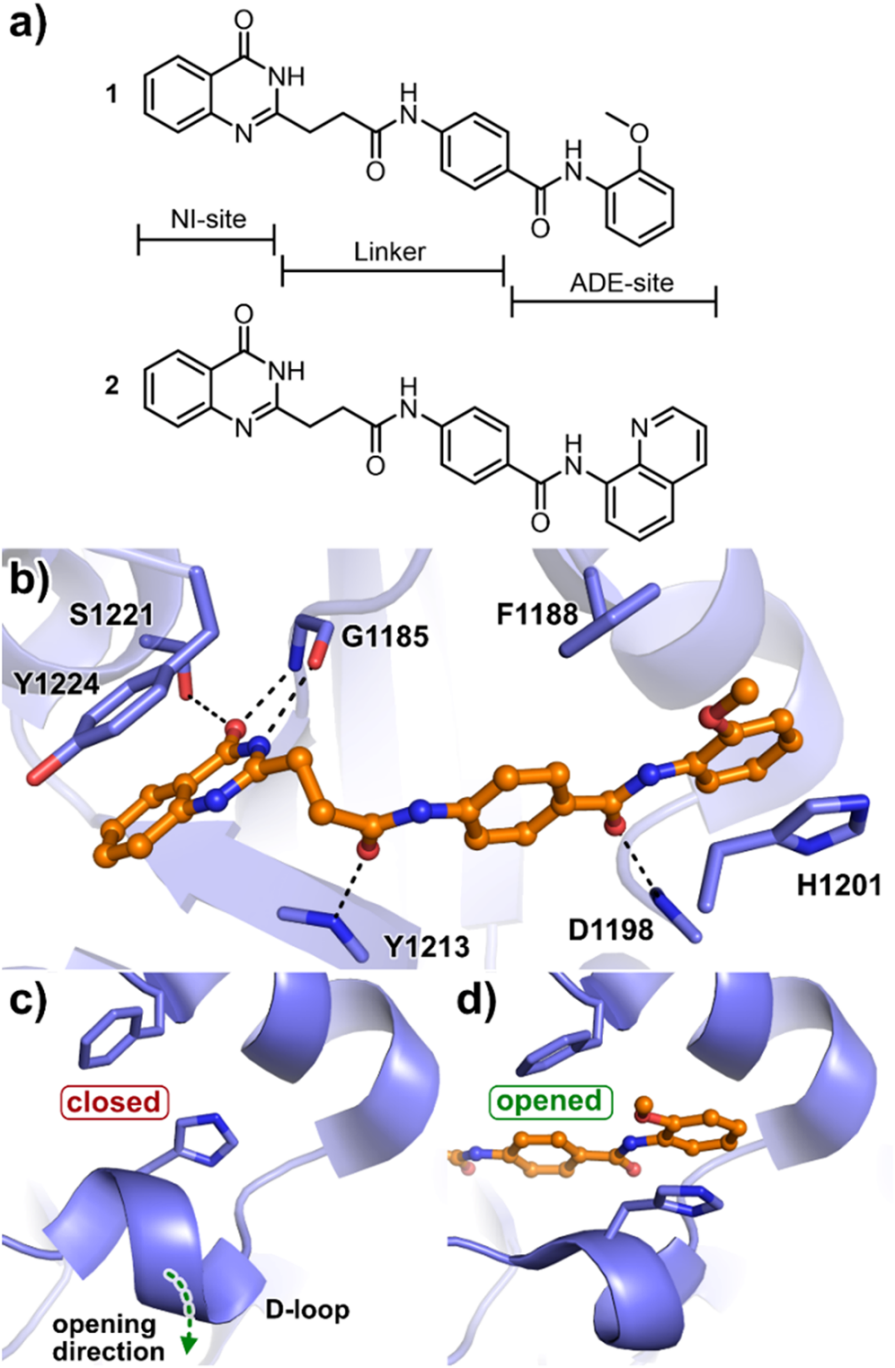
Dual site binders (**1** and **2**) used in the deconstruction analysis. (a) Structure of TNKS, the dual site binders **1** and **2**. (b) Crystal structure of TNKS1 in complex with **1** (PDB: 4I9I). (c) The ADE site of TNKSs is closed in apo-crystal structures (PDB: 3KR7). (d) Ligands that bind to the ADE-site open this subpocket (TNKS1 in complex with **1**, PDB: 4I9I) through a conformational change of the D-loop (dashed arrow in (c)).

Although conflicting observations have been made regarding the extent to which a cryptic ADE pocket serves as a binding hotspot and its contribution to the binding affinity for dual site inhibitors, no systematic deconstruction analysis has been reported in this setting.^19,24−29^ Moreover, the unusually long near-linear expansion of dual inhibitors across the 20 Å long binding cleft raised the question of which specific region of the ligand acts as a linchpin for affinity, to what extent, and which regions exhibit weaker contributions.^29,30^ Here, we explored the fragment deconstruction of two cognate high-affinity TNKS inhibitors, **1** and **2**, with well-defined dual-site binding modes to TNKS1/2, elucidated by X-ray crystallography of the TNKS1-ligand **1**(31) and TNKS2-ligand **2**(32) complex. Both showed similar binding geometries and contacts (Figure 1). These model ligands allowed us to dissect the energetic impact and the role of different moieties, including the linker. From these models, we subsequently propose improved alternative structures deduced in our deconstruction study.

## Results and Discussion

**Figure 2:**
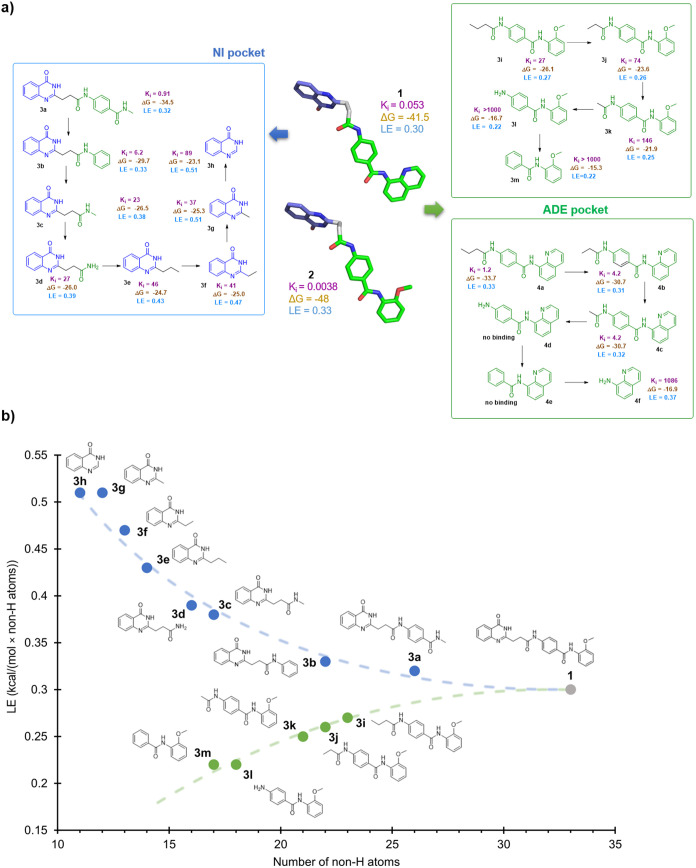
(a) Schematic representation of the deconstruction logic and resulting binding energetics of the fragments for **1** and **2** to TNKS2 [*K*_i_ values of TNKS2 inhibition are given in μM. Δ*G* values are given in kJ/mol; they were calculated at 298 K. Ligand efficiencies (LEs) are given in kcal/(mol × number of non-H atoms)]. (b) Plot of LE against number of non-hydrogen atoms for fragments **3a**–**3h** and **3i–3m**.

We begin our discussion and analysis by inspecting the binding mode of the full inhibitors **1** and **2**. The quinazolin-4(3*H*)-one motif binds to the nicotinamide (NI) pocket via three essential hydrogen bonds, whereas the *p*-aminobenzamide moiety binds to the ADE pocket. These motifs are linked by a flexible propyl amide chain, allowing the NI- and ADE-directed fragments to adopt an optimal interaction geometry (Figure 1a,b). Inhibitors **1** and **2** are similar with respect to their overall structure, except that the *o*-methoxyphenyl group in **1** is replaced by a larger 8-quinoline in **2**. This enables the detection of subtle differences in binding interactions to the induced ADE pocket. Next, experimental deconstruction of the dual binder inhibitors **1** and **2** revealed insights into the affinity contributions and the impact of the linker on Δ*G* and the ligand efficiency (LE) (Table 1, Figure 2). Ligands **1** and **2** were successively dissected using a NI- and ADE-directed deconstruction to obtain smaller fragments, which were then synthesized. We tested the inhibition of TNKS2 using a well-validated standard biochemical assay based on the consumption of the substrate NAD^+^.

**Table 1:**
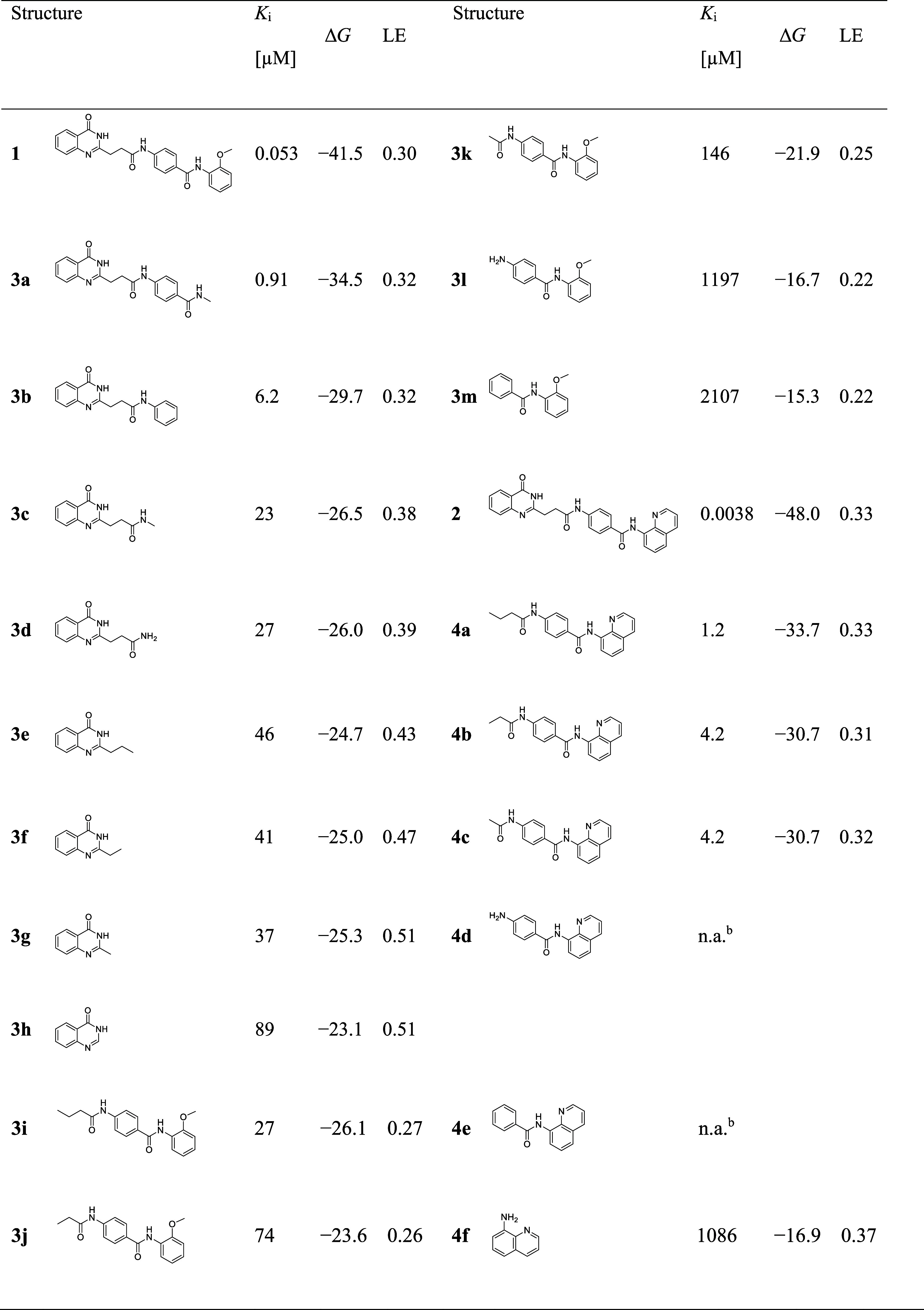
Binding Affinity, Δ*G*, and LE of the Deconstructed Fragments of the TNKS2 Inhibitors **1** and **2**^a^

a *K*_i_ for TNKS2 inhibition values are given in μM. Δ*G* values are given in kJ/mol; they were calculated at 298 K. Ligand efficiencies (LEs) are given in kcal/(mol × number of non-H atoms).

b n.a.: inactive fragments.

TNKS1 and TNKS2 are highly homologous, with 89% sequence identity of the catalytic domain and differences outside the binding pockets.^33,34^ The affinities reported for the small molecule tankyrase inhibitors consistently follow the same ranking order for both isoforms.^35^ Fragments **3a**–**3h** were designed to bind to the NI pocket, whereas fragments **3i**–**3m** and **4a**–**4e** bind to the ADE pocket of TNKS2 (Figure 1, Table 1). The fragments addressing the NI-pocket exhibited a rather limited loss of affinity ranging from a *K*_i_ of 0.91 μM for **3a** to that of 89 μM for the quinazolin-4(3*H*)-one fragment **3h** as the minimal pharmacophoric element. By using LE metrics to normalize the energetic contribution to every heavy atom present in the fragment, we observed an increasing LE from 0.32 for **3a** to 0.51 for the smallest fragment **3h**. This indicates optimal binding of the privileged quinazolin-4(3*H*)-one. The binding of **3b** was surprisingly low, considering the highly similar structural shape to the full inhibitor **1** that preserves the main contacts of the interaction pattern. However, this observation underscores the significance of a hydrogen bond and π–π stacking to D1198/D1045 (TNKS1/TNKS2) and H1201/H1048 (TNKS1/TNKS2). While decreasing LEs are commonly encountered when fragments are grown to full inhibitor,^36^ we observed an opposite trend when we directed deconstruction toward the ADE pocket. Here, in stark contrast to the LE increase for the NI-directed deconstruction of **1**, we did not observe a similar trend in the LE of fragments **3i**–**3m** (Figure 2) accommodating in the ADE pocket. The two smallest fragments, **3l** and **3m**, exhibited the lowest LE values. Growing ADE-directed fragments from **3m** to **3i** and **1** resulted in an expected increase in *K*_i_, while the overall LE successively increased from 0.22 to 0.3 (**3m** < **3i** < **1**). This was in contrast to the observed LE trend of the NI-directed fragments **3a**–**3h**, where smaller fragments resulted in higher LE values. Next, deconstruction of the dual-site inhibitor **2** was performed starting from the left side of the molecule as deconstruction from the other side of the molecule gave rise to the fragments **3a**–**3h**, which were already tested against TNKS2. Here, 8-aminoquinoline (**4f**), as well as the next smallest fragment **4e**, exhibited significantly lower activity. Similar to fragments **3h**–**3k**, *K*_i_ values of fragments **4a**–**4e** increased with an increase in the number of heavy atoms. However, we observed an opposing trend in the NI-directed fragments and LE values of the N-acylated fragments, **4a**–**4c**, were in the range of 0.31–0.33 with no clear pattern. We attributed these unexpectedly high values to the different binding modes of these fragments.^37−41^ Moreover, when we compared the two series of fragments that mainly accommodated the induced ADE pocket of TNKS2, the quinoline fragments **4a**–**4e** showed a stronger affinity than the *o*-methoxyphenyl fragments **3h**–**3k**.

Nevertheless, the consistent trend that fragments targeting the ADE pocket do not exhibit a successively increasing LE after deconstruction could be validated by a similar analysis for a different ligand **3** (Supporting Information, Table S1), which exclusively targets the ADE-site of TNKS2.^42^ Using this structurally distinct ligand, we observed a successive loss of LE when deconstructing the full ADE-site binding inhibitor. Similarly, ADE-directed deconstruction of **2** confirmed the loss of LE toward smaller fragments, particularly when the critical acetamide moiety as a hydrogen bond donor at the linker was absent.

Next, we analyzed the role of the linker contribution from the two deconstructed complementary fragments, recapitulating the energetics of the full inhibitor. In general, a connection of two molecular fragments can affect Δ*G* in several ways. The loss of rigid body translational and rotational entropies is a major hurdle for ligand binding. The entropy loss for the small fragments was estimated to be 15–20 kJ/mol; however, for the full ligand, this value increased only slightly. Thus, the loss of the rigid body entropy represents a lower barrier for binding of the larger ligands.^43,44^ In contrast, the connection of two fragments leads to the adoption of more strained conformations for each of them and together with a loss of rotational linker entropy contributes to an increase in the overall binding barrier.^45,46^ Compensation by careful linker design and screening, together with the application of the necessary structural adjustments of the fragments, maximizes the positive effect of the linkage. In an ideal case, the “superadditivity,” or overproportional synergistic increase of Δ*G* can be reached.^47^ The nonadditivity for Δ*G* contributions is defined as linker contribution Δ*G*_link_ corresponding to the difference between the fragment affinity and final ligand. Using these fragments, Δ*G* contribution of linkers was calculated by subtracting Δ*G* of each fragment from Δ*G* of the final ligand: Δ*G*_link_ = Δ*G*_final_ – Δ*G*_frag1_ – Δ*G*_frag2_ (Table 2). Fragments that could connect to each other via a single bond were chosen to minimize the effect of structural changes. The linkage of fragment **3h** (Δ*G*_frag1_ = −23.1 kJ/mol) to fragment **3j** (Δ*G*_frag2_ = −23.6 kJ/mol) through a carbon–carbon single bond gave rise to the final ligand **1**. The combination of these two fragments, neglecting any cooperative linker contribution to binding, should produce a ligand with a Δ*G* of at least −46.7 kJ/mol. Since the observed Δ*G*_final_ of final ligand **1** was −41.5 kJ/mol, the energetic penalty of linking the two active fragments **3h** and **3j** was +5.2 kJ/mol. Similarly, connecting fragment **3h** (Δ*G*_frag1_ = −23.1 kJ/mol) to fragment **4b** (Δ*G*_frag2_ = −30.7 kJ/mol) through a carbon–carbon single bond generated ligand **2** with an observed Δ*G*_final_ = −47.4 kJ/mol. Again, the Δ*G*_final_ of ligand **2** was higher than that of the linked fragments **3h** and **4b**, with a Δ*G*_link_ of +5.8 kJ/mol, indicating a negative cooperativity of connecting these two fragments. This unfavorable negative cooperativity was similar in the case of fragments **3g** and **3k** (Δ*G*_link_ = +5.7 kJ/mol) and **3g** and **4c** (Δ*G*_link_ = +8.0 kJ/mol) when they were virtually linked by the formation of a carbon–carbon single bond to form ligand **1** and **2**, respectively. Fragments **3d** and **3m** were also combined in the same manner, by forming a carbon–nitrogen bond, to obtain ligand **1**. This had a moderate positive linker contribution to binding (Δ*G*_link_ = –0.3 kJ/mol), which is far below the Δ*G*_link_ estimation of 15–20 kJ/mol by Murray and Verdonk.^43^

**Table 2:**
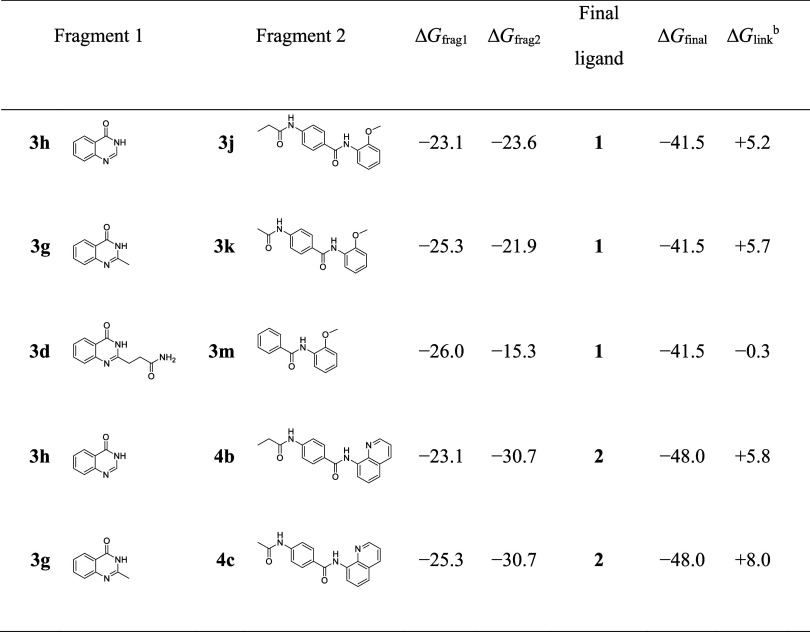
Calculation of Free Energy of Binding Δ*G* Contribution of Linker^a^

a Δ*G* values are given in kJ/mol; they were calculated at 298 K.

b The linker contributions were calculated using the formula Δ*G*_link_ = Δ*G*_final_ – Δ*G*_frag1_ – Δ*G*_frag2_.

The absence of a favorable energetic contribution to the free energy of binding expected from the reduced loss of rigid-body entropy when joining two fragments indicated that the linker of the full inhibitor **1** does not present the essential structural motifs in an optimal geometry. Similar results were obtained after combining the fragment pair **3g** and **4c** to form the final ligand **2**. Consequently, the final full ligands **1** and **2** could not occupy the NI and ADE pockets of TNKS2 as effectively as the individual fragments.

**Figure 3:**
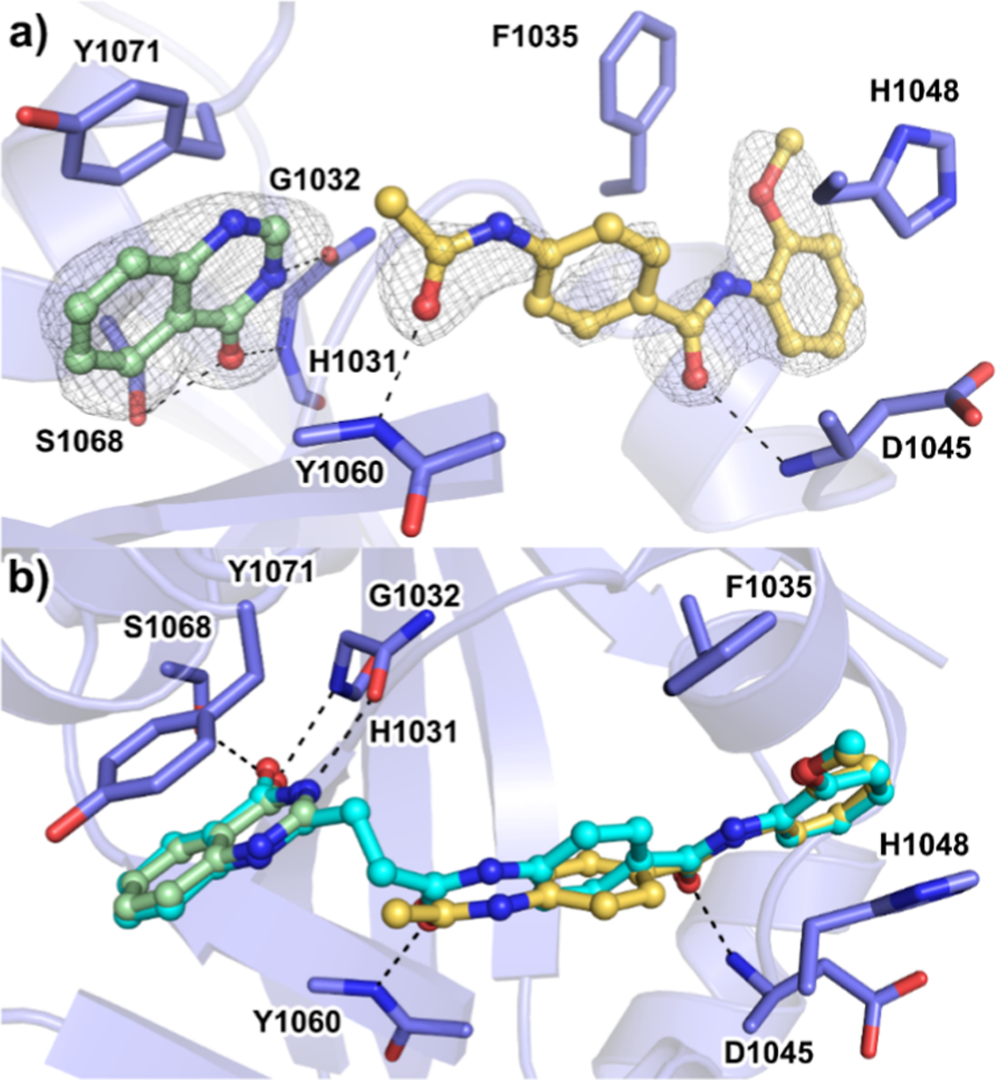
Cocrystal structure of TNKS2 in complex with fragments **3h** and **3k** and comparison of their binding modes with that of the full inhibitor **1**. (a) Binding mode of the fragments **3h** (green) and **3k** (yellow) to the ART (ADP-ribosyltransferase) domain of TNKS2 (PDB: 7OJO) and (b) superimposed structure of **1** (cyan) from a cocrystal structure with TNKS1 (PDB: 4I9I). The protein shown is TNKS2. The σA weighted 2Fo – Fc electron density maps around the ligands are contoured at 1.0 σ.

To confirm and structurally characterize the simultaneous binding of fragments **3h** and **3k** to the catalytic domain of TNKS2 through X-ray crystallography, we utilized a cocrystallization plus soaking approach, where TNKS2 was cocrystallized with **3k**. The crystal was subsequently placed in a solution containing **3h**. The resulting structural model showed the electron densities of both fragments (Figure 3a). Although all hydrogen bonds were conserved and recapitulated the overall binding mode in **3k** compared with **1**, the phenyl-containing linking region of **3k** showed poor electron density, possibly because of the flexibility of this linking region. Similarly, the conformation and position of this linking region differed when comparing the structures of the fragments with **1** (Figure 3b). These results indicate that the linker could be under strain in **1** owing to an unfavorable gauche conformation. A different linker could possibly resolve this strain and result in a higher binding affinity.

### Linker Design

These findings indicated that one of the major structural impairments was the energetically unfavorable linker connection between the fragments that addressed the NI and ADE pockets (Table 2). We conducted a detailed analysis of the ligands based on their 3D superposition, which revealed changes in position due to the linker that twisted the two fragment ends into a less favorable orientation in the binding site. Therefore, we devised an in silico linker optimization approach to design alternative connectors to further investigate the variables that determine the potency of **1**.

First, the ReCore methodology implemented in SeeSAR^48^ was used to enumerate compounds virtually, replacing the linker region at the propyl-amide core of **1** with other suitable linker fragments based on molecules from the public compound library ZINC (ReCore ZINC index 2017).^49^ After this data-driven reconstruction approach the 40 most favorable compounds identified based on the estimated affinity using the built-in Hyde scoring function, were further assessed for their conformational ligand strain. We deprioritized in the ligand selection those molecular designs with nonplanar aromatic ring systems and unfavorable dihedral angles. Eleven compounds were selected for further computational assessment based on their synthetic feasibility (Table S3, compounds **S01**–**S11**). Based on the computational design results, two additional compounds containing cyclobutyl and pentyl moieties were included (Table S3, compounds **S12** and **S13**).

Next, the 13 selected molecules, together with **1** were docked into the binding pocket of the TNKS2 structure cocrystallized with **3h** and 3**k**. Interestingly, docking **1** into the binding site of this TNKS2 structure did not reproduce the binding mode observed in the PDB structure 4I9I,^31^ which further indicates the energetically unfavorable conformation of **1** in TNKS2. Hence, docking was performed using the hybrid method implemented in the OEDocking toolkit 4.1.0.1^50^ by transferring the cocrystallized ligand from the PDB structure 4I9I to bias the ligand placement during docking. Using these settings, the observed binding mode of **1** could be reproduced, and compounds with alternative linkers (**S01**–**S13**) were docked. After energy minimization with the MMFF94 force field and calculation of the binding-affinity score, the docking poses were visually inspected using LigandScout 4.4.^51^

Compound **1** was used as a reference to guide the selection of linker candidates for further investigation. The docking poses of compounds **S01**–**S13** were inspected for the presence of two hydrogen bonds with the backbone of the ADE pocket, which were only observed in compounds **1**, **S02**, **S08**, and **S11**–**S13** (Table S3). One hydrogen bond was observed in **S01**, **S04**–**S07**, **S10**, and **S11**. In addition, the docking poses were manually evaluated for ligand strain characterized by bent aromatic systems or unfavorable bond angles. Docking poses of ligands without strain (**1**, **S01**, **S02**, **S05**, **S06**, **S08**, **S12**, and **S13**) were submitted to MD simulations using OpenMM 7.6^52^ together with the Amber14SB and GAFF2.11 force fields.^53^ For each complex, the trajectory was visually inspected for the stability of the linker in the binding pocket. Only the linkers of **1**, **S02**, **S05**, and **S06** showed a stable position over the course of 100 ns of the MD simulation. Computational evaluation combined with synthetic feasibility assessment led to the selection of **S01**, **S02**, **S06**, and **S13** for chemical synthesis and biological evaluation. Prioritized candidate ligands were synthesized, resulting in six new compounds with unique linkers (compounds **5**–**10**). Compounds **7**, **8**, **9** and **10** were prepared as stereoisomers (Supporting Information).

All the designed compounds with new linkers inhibited TNKS2 with potencies varying between *K*_i_ of 8.5 and 868 nM (Table 3). The small urea-based linker of the compound **5** was well tolerated and showed a potency similar to that of benchmark compound **1** (*K*_*i*_ of 88 and 53 nM, respectively). The addition of methylene thiophene to **6** instead of an unsubstituted methyl showed a significant improvement of over 1 order of magnitude with a *K*_*i*_ of 8.5 nM, in agreement with previous findings.^31^ For the cyclobutyl linker, we synthesized both cis- (**8**) and trans-analogs (**7**), and as predicted by the original design, cis-isomer **8** showed better affinity with a *K*_i_ of 10 nM compared to a *K*_i_ of 868 nM for **7**. For the pyrrolidine linker variants in **9** and **10**, both enantiomers were active with *K*_i_ values of 190 and 34 nM, respectively; however, neither reached the affinity of the best compounds. We next investigated whether the best compounds could inhibit TNKSs in the cellular context as well using a β-catenin reporter assay with HEK293 cells.^12,14,54,55^ The parent compound **1** showed an IC_50_ of 530 ± 280 nM. The tested new analog **6** showed an improved IC_50_ of 71 ± 480 nM in the cellular assay and good correlation with the enzyme assays, whereas **8** showed poor potency of 1300 ± 480 nM, most presumably due to low membrane permeability.

**Table 3:**
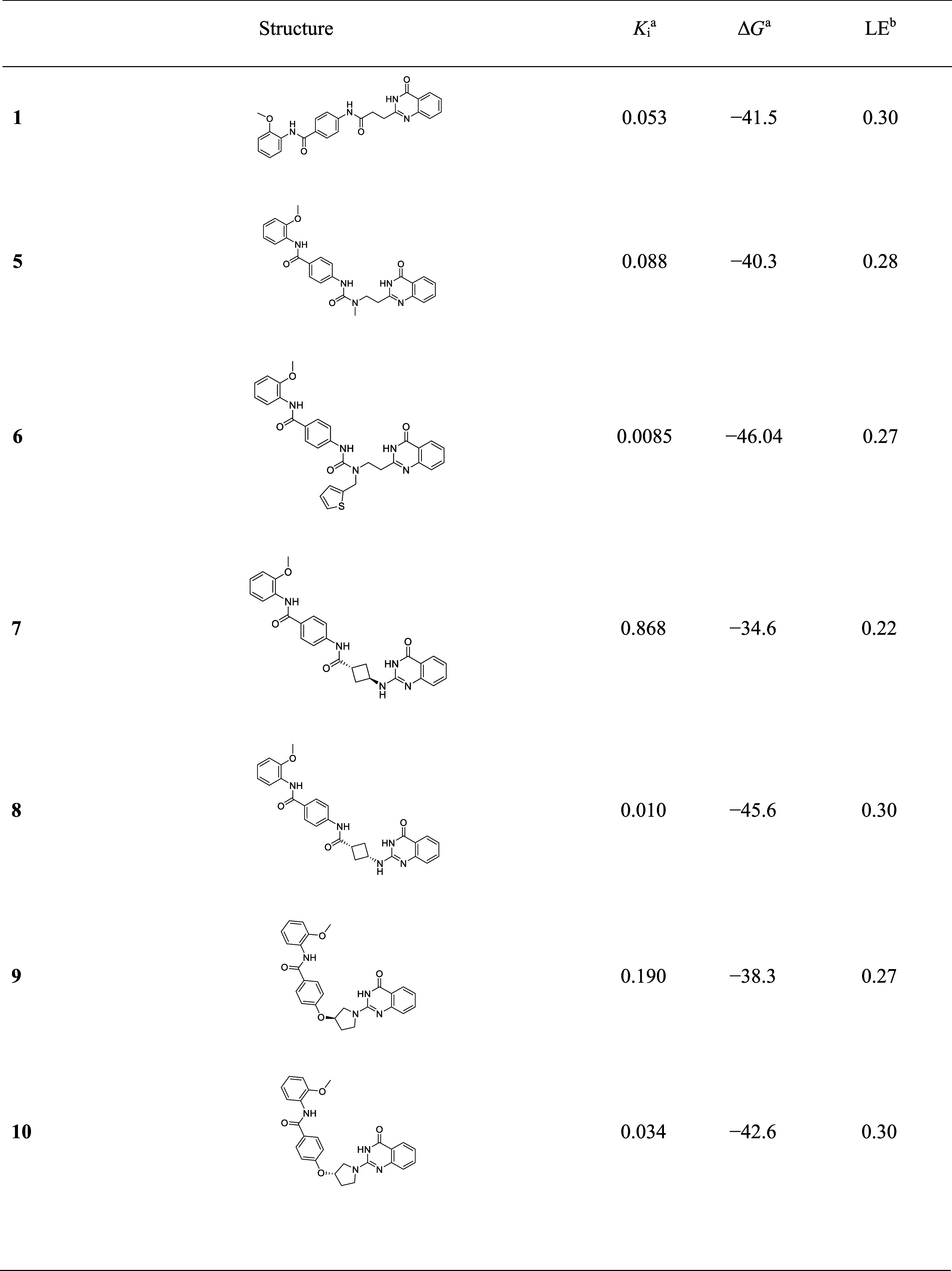
Inhibition of TNKS2 by the Compounds with New Linker Designs

a *K*_i_ for TNKS2 inhibition values are given in μM. Δ*G* values are given in kJ/mol; they were calculated at 298 K.

b Ligand efficiencies (LEs) are given in kcal/(mol × number of non-H atoms).

**Figure 4:**
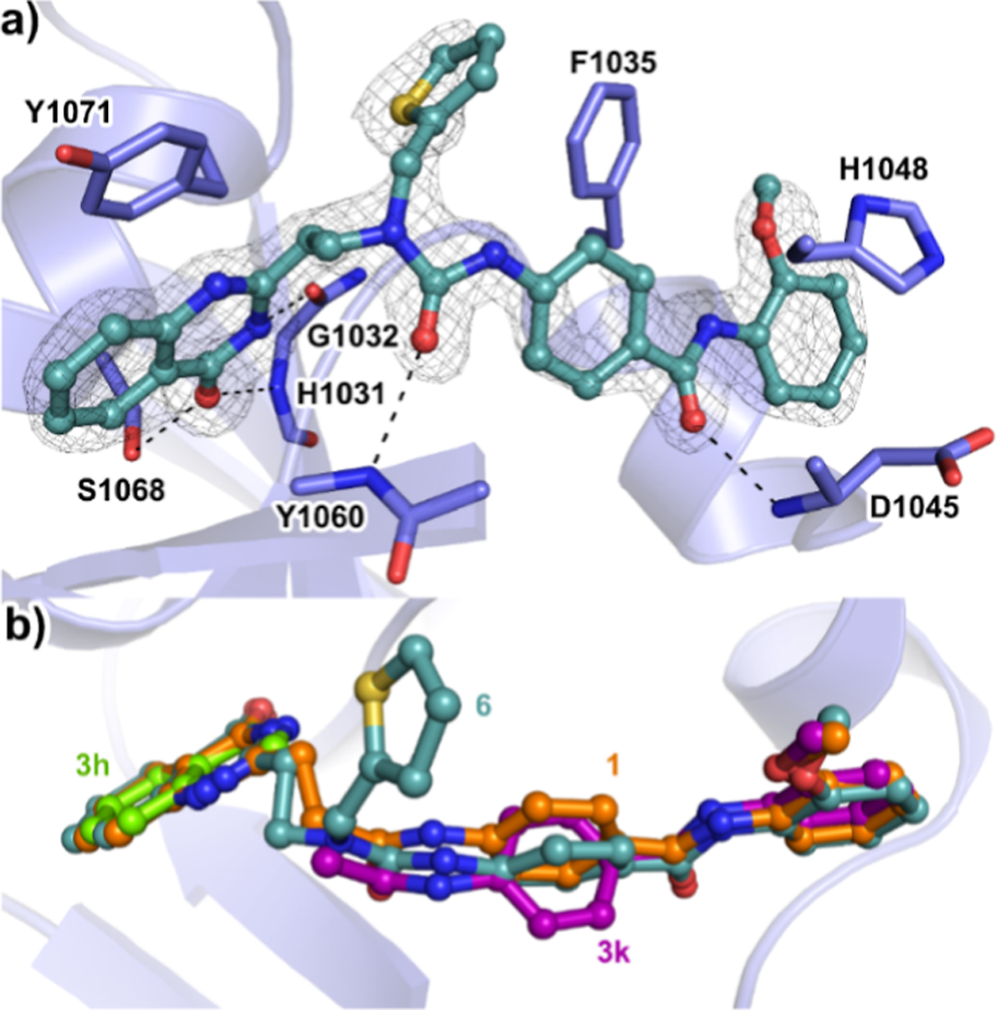
Crystal structure of **6** in complex with TNKS2. (a) Cocrystal structure of TNKS2 in complex with **6**. Side chains involved in binding of the compound are shown. The σA weighted 2Fo – Fc electron density map is contoured at 1.0 σ. (b) Comparison of the binding modes of **1** (PDB: 4I9I), **6** (PDB: 8B6M), and **3k** and **3h** (PDB: 7OJO).

To investigate whether the conformational restraints inherent to the propyl amide linker in **1** were absent in compound **6**, the cocrystal structure of the TNKS2s catalytic domain was solved. The compound was strongly bound, whereas the thiophene moiety appeared to be more flexible, as indicated by its poor electron density (Figure 4a). This compound replicated the binding of the fragments and maintained key hydrogen bonds with the backbone of the protein. However, the central phenyl group rotated to allow adjustments in other regions to relieve strain when the fragments were linked (Figure 4b). This rotation was also evident when comparing the earlier cocrystal structure of **1** with that of TNKS2 (Figure 4b). While the improved potencies confirm our fragment deconstruction analysis, the rotational shift in the experimental structure suggests that the designed linker in **6** could ameliorate the previous strain in **1** to some extent but could not completely eliminate it.^42^

### Conclusions

Although a wide array of TNKS ligands have been reported previously, to the best of our knowledge a systematic fragment-based investigation of TNKS inhibitors addressing their binding energetics and the contribution of individual fragments and linkers to the overall binding affinity has not yet been published before. Here, we investigated the molecular energetic features of a dual-binding ligand addressing a cryptic pocket, through systematic deconstruction of two cognate inhibitors. Deconstruction of the NI site showed that the LE increased with successive fragmentation and even the smallest fragments showed significant affinity. This indicates a strong interaction with the privileged quinazoline moiety. Fragment deconstruction directed toward the ADE site showed the opposite trend, highlighting the essential features of this cryptic pocket. Smaller fragments showed extremely low to negligible binding affinity, resulting in decreased LEs of the smaller fragments compared with the full inhibitor. The decreasing LEs indicate that the present fragments were unable to address a particular hotspot interaction. The affinity contributions of larger fragments arose from a sum of weak collective interactions. Similar observations were made for the second privileged fragment and for the deconstruction of an inhibitor that solely addresses the ADE-binding site. Thus, this might be characteristic of this cryptic pocket, given the considerable energetic penalty for the rearrangement and the absence of a strong interaction hot spot, but also a general hurdle and complication for fragment-based drug discovery addressing cryptic pockets.

Deconstruction analysis further revealed that the present linker geometry misaligned both binding elements at the NI and ADE pocket compared with their deconstructed fragments. This misalignment caused a substantial energetic penalty when both fragments were linked instead of the expected gain of 15–20 kJ/mol, irrespective of whether the *o*-methoxyphenyl or 8-quinoline moiety was analyzed. Inspection of the X-ray structure in which fragments **3h** and **3k** were simultaneously bound to the catalytic domain of TNKS2 confirmed an unfavorable linker geometry, resulting in attenuation of the main contacts. This cocrystal structure prompted us to investigate this region and seek improved linkers to circumvent the unfavorable linker geometry. We successfully identified alternative moieties with significantly higher overall affinities by structure-based computational design and probing several other linker geometries ranging from a flexible amine-base linker, over a urea moiety and a rigid cyclobutane linker. This further validated the deconstruction analysis results. Although highly privileged optimal fragments were used in our deconstruction study, this investigation further showed that the design of an optimal linker is a critical endeavor that requires careful analysis and deserves strong attention in fragment-based drug design.

## Experimental Section

### Protein Production

Production of TNKS2 catalytic domain for crystallization was done as previously described.^56^ For biochemical assays, TNKS2 containing the SAM and catalytic domain was used. The sequence of TNKS2 (SAM and catalytic domain, residues 873–1162) was cloned into a pNIC-MBP vector, encoding for a TEV cleavable N-terminal His_6_-MBP tag.^57^ Chemically competent *Escherichia coli* BL21(DE3) cells were transformed with the plasmid and plated on LB agar containing 50 μg/mL kanamycin. A preculture from a single colony was incubated at 37 °C for 18 h and used to inoculate (1:100) 2 L of TB autoinduction media including trace elements (ForMedium), supplemented with 8 g/L glycerol and 50 μg/mL kanamycin. The culture was subdivided into four 5 L shaking flasks and incubated at 37 °C in a shaker until an OD of 0.6–1.0 was reached. The temperature was lowered to 18 °C and incubation in the shaker continued for 20 h. The cells were harvested by centrifugation at 4200*g* for 10 min. The pellet was resuspended in 80 mL lysis buffer (50 mM HEPES pH 7.5, 500 mM NaCl, 0.5 mM TCEP, 10 mM imidazole, 10% glycerol) and stored at −20 °C. For purification, cells were thawed and lysed by sonication. The lysate was centrifuged (16 000*g*, 4 °C, 30 min), filtered and loaded onto a 5 mL HiTrap HP column (Cytiva) equilibrated with lysis buffer and charged with Ni^2+^. The column was washed with five column volumes of lysis buffer and five column volumes of lysis buffer containing 25 mM imidazole. The protein was eluted using lysis buffer containing 300 mM imidazole. Elutions were directly loaded to a 5 mL MBPTrap HP column (Cytiva). The column was washed with five column volumes 20 mM HEPES pH 7.5, 200 mM NaCl, 0.5 mM TCEP and eluted using the same buffer including 10 mM maltose. Higher activity of the construct was observed without the MBP-tag. The protein was therefore incubated with TEV protease (1:10 molar ratio) for 48 h at 4 °C to remove the MBP-tag. The protein was flash frozen in liquid nitrogen and stored at −70 °C.

### Biochemical Tankyrase Assay

The enzymatic assay measures unreacted NAD^+^, which is chemically converted into a fluorescent compound.^58^ The fluorescence intensity was measured with excitation/emission wavelengths of 372 and 444 nm, respectively, using Tecan Infinity M1000 Pro. New compounds were prepared in half-log dilution series, and the reactions were carried out in quadruplicates in 384-well plates (black, Fisherbrand ShallowWell) with protein and compound controls to exclude the effect of compound autofluorescence. Compounds dissolved in DMSO were dispensed using an Echo 650 acoustic dispenser (Beckmann Coulter) and assay reagents were dispensed using a Mantis liquid dispenser (Formulatrix). All reactions were performed at ambient temperature (22 °C). TNKS2 protein (SAM and catalytic domain, 20 nM) was incubated for 20 h in assay buffer (50 mM Bis-Tris propane (BTP), pH 7.0, 0.5 mM Tris(2-carboxyethyl)phosphine (TCEP), 0.01% Triton X-100) with compound and 5 μM NAD^+^, resulting in approximately 50% conversion of the NAD^+^ in the controls excluding the compound. All conditions contained 1% DMSO. For each compound, three independent IC_50_ curves were measured. The geometric mean of the IC_50_ values was used to calculate the *K*_i_ values.

### General Information

Reactions requiring anhydrous conditions were carried out in dry solvents from Sigma-Aldrich stored over molecular sieves which were used as received. Purification of compounds was performed by flash chromatography on silica using a Biotage Isolera One apparatus or by HPLC using a Gilson PLC-2050 system equipped with a UV/visible and ELSD detectors, and a Nucleodur 100-7 C18ec column by Marchey-Nagel. Water/acetonitrile with 0.1% TFA was used as the solvent system, with the specified methods. Lyophilization was performed with the Lyophilizer ALPHA 2-4 LSC from Christ.

NMR spectra were acquired on devices from the company Bruker (AV 300, AV 600). All ^13^C NMR-spectra were recorded with ^1^H-broad-band decoupling. Calibration of the chemical shift was conducted using the solvent residual signals. Numbering of the denoted compounds arises from the IUPAC nomenclature. Mass spectra were recorded with an Agilent 1260 liquid chromatography coupled accurate mass time-of-flight 6230 detector. Standard analysis of reaction time courses was conducted with an Agilent 1260 infinity liquid chromatography coupled quadrupole mass spectrometer 6120 detector.

Compound purity was ensured HPLC measurement and exceeded 95% purity for all tested compounds.

#### General Procedure A: Amide Coupling

Step 1: DMAP (0.1 equiv) and EDCI (1.2 equiv) were sequentially added to a mixture of 4-*tert*-butoxycarbonylbenzoic acid (1.0 equiv) and heterocycle (1.0 equiv) in dry DCM/DMF 15:1 mixture (0.15 M) under N_2_ atmosphere. The mixture was stirred at room temperature for 14 h. The reaction was quenched by addition of water, and aqueous layer was extracted three times with DCM. Combined organic layers were washed with 0.1 M HCl and brine. The crude was dried over Na_2_SO_4_ and concentrated in vacuum. The products were used as a crude, purified by recrystallization or flash chromatography.

Step 2: the product of step 1 was dissolved in DCM/TFA (4:1) and a mixture was stirred for 2 h. The solvent was removed by coevaporation with toluene in vacuum, and the crude was purified by recrystallization or flash chromatography.

#### General Procedure B: *N*-Acylation

Triethylamine (1.2 equiv) and acyl chloride (1.2 equiv) were sequentially added to aromatic amine (1.0 equiv) in dry DCM (0.15 M) under N_2_ atmosphere. The mixture was stirred at room temperature for 3–12 h. The reaction was quenched by addition of water, and aqueous layer was extracted three times with DCM. Combined organic layers were washed with 0.1 M HCl and brine. The crude was dried over Na_2_SO_4_ and concentrated in vacuum. The products were used as a crude, purified by recrystallization or flash chromatography.

##### 3-(4-Oxo-3,4-dihydroquinazolin-2-yl)propanoic Acid

Methyl 4-chloro-4-oxobutanoate (1.00 mL, 8.08 mmol, 1.1 equiv) was slowly added to a solution of 2-aminobenzamide (1.00 g, 7.34 mmol, 1.0 equiv) and pyridine (0.77 mL, 9.55 mmol, 1.3 equiv) in anhydrous THF (25 mL) at 0 °C under nitrogen atmosphere. The reaction mixture was stirred at this temperature for 10 min and then overnight at room temperature. It was quenched with saturated NaHCO_3_ solution (40 mL) and extracted three times with dichloromethane (50 mL). Combined organic extracts were dried over MgSO_4_, filtered and all volatiles were evaporated under vacuum. The colorless solid (1.61 g, 88% yield) was used as crude in the next step without further purification. The crude was then added to a 0.5 M of NaOH solution (300 mL). The mixture was stirred for 1 h at 60 °C. After cooling it down, the mixture was acidified to pH 2 by adding HCl solution (2 M). The volume was reduced to 100 mL under vacuum. The precipitate was collected by filtration and washed with water and small amount of ethanol to obtain the title compound (500 mg, 48% yield) as an amorphous solid.

^1^H NMR (300 MHz, DMSO-*d*_6_): δ 12.74 (br s, 2H), 8.20–8.09 (m, 1H), 8.00–7.85 (m, 2H), 7.63 (ddd, *J* = 8.2, 6.7, 1.7 Hz, 1H), 3.08 (t, *J* = 7.4 Hz, 2H), 2.95 (t, *J* = 7.4 Hz, 2H) ppm.

Characterization was consistent with the previously published values.^59^

##### *N*-Methyl-4-(3-(4-oxo-3,4-dihydroquinazolin-2-yl)propanamido)benzamide (**3a**)

4-Amino-*N*-methylbenzamide (83 mg, 1.2 equiv) was added to a suspension of 3-(4-oxo-3,4-dihydroquinazolin-2-yl)propanoic acid (100 mg, 0.46 mmol, 1.0 equiv) and HATU (93 mg, 0.55 mmol, 1.2 equiv) in anhydrous DMF (4.6 mL). The mixture was stirred overnight. It was then diluted with DCM (10 mL) and quenched with water (5 mL). The phases were separated and the aqueous phase was extracted twice with DCM (10 mL). Combined organic phases were dried over MgSO_4_, filtered and concentrated under vacuum. The crude product was further purified by column chromatography to obtain compound **3a** (33 mg, 21% yield) as an amorphous solid.

^1^H NMR (300 MHz, DMSO-*d*_6_): δ 12.26 (br s, 1H), 10.29 (br s, 1H), 8.30 (q, *J* = 4.0 Hz, 1H), 8.09 (dd, *J* = 8.0, 1.5 Hz, 1H), 7.81–7.72 (m, 3H), 7.68–7.61 (m, 2H), 7.55 (d, *J* = 8.0 Hz, 1H), 7.51–7.42 (m, 1H), 3.03–2.85 (m, 4H), 2.76 (d, *J* = 4.5 Hz, 3H) ppm.

^13^C NMR (151 MHz, DMSO-*d*_6_): δ 170.92, 166.54, 162.07, 156.98, 149.18, 142.12, 134.76, 129.29, 128.32, 127.19, 126.45, 126.20, 121.38, 118.56, 32.78, 29.62, 26.64 ppm.

HRMS (pos. ESI-TOF): *m*/*z* calcd for C_19_H_18_N_4_O_3_ 373.1277 [M + Na]^+^; found, 373.1275 [M + Na]^+^.

##### 3-(4-Oxo-3,4-dihydroquinazolin-2-yl)-*N*-phenylpropanamide (**3b**)

Aniline (51 mg, 0.55 mmol, 1.2 equiv) was added to a suspension of 3-(4-oxo-3,4-dihydroquinazolin-2-yl)propanoic acid (100 mg, 0.46 mmol, 1.0 equiv) and HATU (93 mg, 0.55 mmol, 1.2 equiv) in anhydrous DMF (4.6 mL). The mixture was stirred overnight. It was then diluted with DCM (10 mL) and quenched with water (5 mL). The phases were separated and the aqueous phase was extracted twice with DCM (10 mL). Combined organic phases were dried over MgSO_4_, filtered and concentrated under vacuum. The crude product was further purified by column chromatography to obtain compound **3b** (37 mg, 28% yield) as an amorphous solid.

^1^H NMR (300 MHz, DMSO-*d*_6_): δ 12.25 (s, 1H), 10.08 (s, 1H), 8.08 (dd, *J* = 7.9, 1.6 Hz, 1H), 7.81–7.70 (m, 1H), 7.62–7.51 (m, 3H), 7.45 (t, *J* = 7.5 Hz, 1H), 7.30–7.25 (m, 2H), 7.01 (t, *J* = 7.3 Hz, 1H), 3.01–2.80 (m, 4H) ppm. ^13^C NMR (151 MHz, DMSO-*d*_6_): δ 170.53, 162.21, 157.20, 149.17, 139.74, 134.70, 129.12, 127.11, 126.38, 126.20, 123.41, 121.36, 119.44, 32.81, 29.83 ppm.

HRMS (pos. ESI-TOF) *m*/*z* calcd for C_17_H_15_N_3_O_2_ 316.1061 [M + H]^+^; found, 316.1071 [M + H]^+^.

##### *N*-Methyl-3-(4-oxo-3,4-dihydroquinazolin-2-yl)propanamide (**3c**)

A methylamine solution (2 M in THF, 0.7 mL, 1.37 mmol, 3.0 equiv) was added to a suspension of 3-(4-oxo-3,4-dihydroquinazolin-2-yl)propanoic acid (100 mg, 0.46 mmol, 1.0 equiv) and HATU (100 mg, 0.60 mmol, 1.3 equiv) in anhydrous DMF (4.6 mL). The mixture was stirred overnight. It was then diluted with DCM (10 mL) and quenched with water (5 mL). The phases were separated and the aqueous phase was extracted twice with DCM (10 mL). Combined organic phases were dried over MgSO_4_, filtered and concentrated under vacuum. The crude product was further purified by column chromatography to obtain compound **3c** (14 mg, 13% yield) as an amorphous solid.

^1^H NMR (300 MHz, DMSO-*d*_6_): δ 12.20 (br s, 1H), 8.07 (dd, *J* = 7.9, 1.6 Hz, 1H), 7.87 (q, *J* = 4.8 Hz, 1H), 7.76 (td, *J* = 7.9, 7.1, 1.6 Hz, 1H), 7.56 (d, *J* = 7.1 Hz, 1H), 7.45 (t, *J* = 7.5 Hz, 1H), 2.87–2.79 (m, 2H), 2.62–2.54 (m, 5H) ppm.

HRMS (pos. ESI-TOF): *m*/*z* calcd for C_12_H_13_N_3_O_2_ 232.1086 [M + H]^+^; found, 32.1082 [M + H]^+^.

##### 3-(4-Oxo-3,4-dihydroquinazolin-2-yl)propanamide (**3d**)

A concentrated ammonia solution (0.26 mL, 8.0 equiv) was added to a suspension of 3-(4-oxo-3,4-dihydroquinazolin-2-yl)propanoic acid (100 mg, 0.46 mmol, 1.0 equiv) and HATU (154 mg, 0.92 mmol, 2.0 equiv) in anhydrous DMF (4.6 mL). The mixture was stirred overnight. It was then diluted with DCM (10 mL) and quenched with water (5 mL). The phases were separated and the aqueous phase was extracted twice with DCM (10 mL). Combined organic phases were dried over MgSO_4_, filtered and concentrated under vacuum. The crude product was further purified by column chromatography to obtain compound **3d** (57 mg, 57% yield) as an amorphous solid.

^1^H NMR (300 MHz, DMSO-*d*_6_): δ 12.13 (br s, 1H), 8.07 (dd, *J* = 8.0, 1.6 Hz, 1H), 7.76 (td, *J* = 8.0, 8.0, 1.6 Hz, 1H), 7.56 (d, *J* = 8.0 Hz, 1H), 7.51–7.32 (m, 2H), 6.84 (s, 1H), 2.82 (t, *J* = 7.4 Hz, 2H), 2.59 (t, *J* = 7.4 Hz, 2H) ppm.

^13^C NMR (75 MHz, DMSO-*d*_6_): δ 173.14, 161.67, 156.88, 148.82, 134.30, 126.78, 125.97, 125.74, 120.90, 31.14, 29.62.

HRMS (pos. ESI-TOF): *m*/*z* calcd for C_11_H_11_N_3_O_2_ 218.0929 [M + H]^+^; found, 218.0936 [M + H]^+^.

##### 2-Propylquinazolin-4(3*H*)-one (**3e**)

Butyryl chloride (274 μL, 2.64 mmol, 1.2 equiv) was slowly added to a solution of 2-aminobenzamide (300 mg, 2.20 mmol, 1.0 equiv) and pyridine (249 μL, 3.08 mmol, 1.4 equiv) in anhydrous THF (11 mL) at 0 °C under nitrogen atmosphere. The reaction mixture was stirred at this temperature for 10 min and then overnight at room temperature. It was quenched with saturated NaHCO_3_ solution (15 mL) and extracted three times with dichloromethane (25 mL). Combined organic extracts were dried over MgSO_4_, filtered and all volatiles were evaporated under vacuum. The colorless solid was used as crude in the next step without further purification. The crude was then added to a 0.5 M of NaOH solution (30 mL). The mixture was stirred for 1 h at 60 °C. After cooling it down, the mixture was acidified to pH 4 by adding HCl solution (2 M). The precipitate was collected by filtration and washed with water to obtain compound **3e** (138 mg, 36% yield over two steps) as an amorphous solid.

^1^H NMR (300 MHz, DMSO-*d*_6_): δ 12.17 (s, 1H), 8.07 (dd, *J* = 8.0, 1.6 Hz, 1H), 7.76 (ddd, *J* = 8.5, 7.1, 1.6 Hz, 1H), 7.62–7.54 (m, 1H), 7.45 (ddd, *J* = 8.0, 7.1, 1.2 Hz, 1H), 2.57 (dd, *J* = 8.4, 6.7 Hz, 2H), 1.74 (h, *J* = 7.4 Hz, 2H), 0.93 (t, *J* = 7.4 Hz, 3H) ppm.

^13^C NMR (75 MHz, DMSO-*d*_6_): δ 162.29, 157.77, 149.41, 134.74, 127.27, 126.38, 126.14, 121.26, 36.81, 20.67, 13.98 ppm.

HRMS (pos. ESI-TOF): *m*/*z* calcd for C_11_H_12_N_2_O 211.0847 [M + Na]^+^; found, 211.0849 [M + Na]^+^.

##### 2-Ethylquinazolin-4(3*H*)-one (**3f**)

Propionyl chloride (154 μL, 1.76 mmol, 1.2 equiv) was slowly added to a solution of 2-aminobenzamide (200 mg, 1.47 mmol, 1.0 equiv) and pyridine (166 μL, 2.06 mmol, 1.4 equiv) in anhydrous THF (6 mL) 0 °C under nitrogen atmosphere. The reaction mixture was stirred at this temperature for 10 min and then overnight at room temperature. It was quenched with saturated NaHCO_3_ solution (10 mL) and extracted three times with dichloromethane (15 mL). Combined organic extracts were dried over MgSO_4_, filtered and all volatiles were evaporated under vacuum. The colorless solid was used as crude in the next step without further purification. The crude was then added to a 0.5 M of NaOH solution (7.3 mL). The mixture was stirred for 1 h at 60 °C. After cooling it down, the mixture was acidified to pH 4 by adding HCl solution (2 M). The precipitate was collected by filtration and washed with water to obtain compound **3f** (125 mg, 49% yield over two steps) as an amorphous solid.

^1^H NMR (300 MHz, DMSO-*d*_6_): δ = 12.16 (s, 1H), 8.07 (dd, *J* = 8.1, 1.6 Hz, 1H), 7.76 (ddd, *J* = 8.5, 7.1, 1.6 Hz, 1H), 7.59 (d, *J* = 8.1 Hz, 1H), 7.51–7.35 (m, 1H), 2.62 (q, *J* = 7.5 Hz, 2H), 1.24 (t, *J* = 7.5 Hz, 3H) ppm.

Characterization was consistent with the previously published values.^60^

##### 2-Methylquinazolin-4(3*H*)-one (**3g**)

Acetyl chloride (126 μL, 1.76 mmol, 1.2 equiv) at 0 °C was slowly added to a solution of 2-aminobenzamide (200 mg, 1.47 mmol, 1.0 equiv) and pyridine (166 μL, 2.06 mmol, 1.4 equiv) in anhydrous THF (6 mL) under nitrogen atmosphere. The reaction mixture was stirred at this temperature for 10 min and then overnight at room temperature. It was quenched with saturated NaHCO_3_ solution (10 mL) and extracted three times with dichloromethane (15 mL). Combined organic extracts were dried over MgSO_4_, filtered and concentrated down under vacuum. The colorless solid was used as crude in the next step without further purification. The crude was then added to a 0.5 M of NaOH solution (7.3 mL). The mixture was stirred for 1 h at 60 °C. After cooling it down, the mixture was acidified to pH 4 by adding HCl solution (2 M). The precipitate was collected by filtration and washed with water to obtain compound **3g** (45 mg, 19% yield over two steps) as an amorphous solid.

^1^H NMR (300 MHz, DMSO-*d*_6_): δ 8.16 (d, *J* = 7.9 Hz, 1H), 7.96 (t, *J* = 7.8 Hz, 1H), 7.83 (d, *J* = 8.2 Hz, 1H), 7.65 (t, *J* = 7.7 Hz, 1H), 2.61 (s, 3H) ppm.

Characterization was consistent with the previously published values.^60^

##### *N*-(2-Methoxyphenyl)-4-nitrobenzamide

A solution of 4-nitrobenzoic acid chloride (1.63 g, 9.74 mmol) in dichloromethane (5 mL) was added dropwise to a solution of *o*-anisidine (0.916 mL, 8.12 mmol) and pyridine (0.786 mL, 9.74 mmol) in dichloromethane (5 mL). The mixture was stirred at room temperature for 18 h. The reaction was quenched by addition of water, and aqueous layer was extracted three times with DCM. Combined organic layers were washed successively with a saturated solution of sodium bicarbonate, 1 M HCl and brine. The crude was dried over Na_2_SO_4_ and concentrated under vacuum affording the title compound (2.05 g, 93%). The product was used in the subsequent reactions without further purification.

^1^H NMR (300 MHz, CDCl_3_): δ 8.60 (s, 1H), 8.51 (dd, *J* = 8.0, 1.7 Hz, 1H), 8.40–8.32 (m, 2H), 8.11–8.03 (m, 2H), 7.16 (td, *J* = 7.8, 1.7 Hz, 1H), 7.06 (td, *J* = 7.8, 1.5 Hz, 1H), 6.97 (dd, *J* = 8.1, 1.5 Hz, 1H), 3.97 (s, 3H) ppm. Characterization was consistent with the previously published values.^6^

##### 4-Butyramido-*N*-(2-methoxyphenyl)benzamide (3i)

4-Butyramido-*N*-(2-methoxyphenyl)benzamide was synthesized according to general procedure B using 4-amino-*N*-(2-methoxyphenyl)benzamide (100.0 mg, 0.41 mmol), TEA (69.0 μL, 0.49 mmol), butyryl chloride (0.49 mmol, 50 μL) and 3 mL of DCM. The crude was purified by flash chromatography (SiO_2_, cyclohexane/EtOAc 1:0 to 1:1) to obtain compound **3i** in 73% yield (94 mg, 0.24 mmol, white powder).

^1^H NMR (300 MHz, CDCl_3_): δ 8.62–8.39 (m, 2H), 7.85 (d, *J* = 8.3 Hz, 2H), 7.67 (d, *J* = 8.4 Hz, 2H), 7.69–7.56 (bm, 1H), 7.13–6.94 (m, 2H), 6.92 (d, *J* = 8.0 Hz, 1H), 3.92 (s, 3H), 2.37 (t, *J* = 7.5 Hz, 2H), 1.76 (q, *J* = 7.4 Hz, 2H), 1.00 (t, *J* = 7.5 Hz, 3H) ppm.

^13^C NMR (75 MHz, CDCl_3_): δ 171.83, 164.85, 148.29, 141.43, 130.50, 128.26 (2C), 127.84, 124.02, 121.28, 119.94 (2C), 119.42, 110.06, 55.96, 39.79, 19.08, 13.88 ppm.

HRMS (pos. ESI-TOF): *m*/*z* calcd for C_18_H_20_N_2_O_3_ 313.1547 [M + H]^+^; found, 313.1563 [M + H]^+^.

##### *N*-(2-Methoxyphenyl)-4-propionamidobenzamide (**3j**)

*N*-(2-Methoxyphenyl)-4-propionamidobenzamide was synthesized according to general procedure B using 4-amino-*N*-(2-methoxyphenyl)benzamide (100.0 mg, 0.41 mmol), TEA (69.0 μL, 0.49 mmol), propionyl chloride (0.49 mmol, 44 μL) and 3 mL of DCM. The crude was purified by flash chromatography (SiO_2_, cHex/EtOAc 1:0 to 1:1) to obtain compound **3j** in 58% yield (72 mg, 0.24 mmol).

^1^H NMR (300 MHz, CDCl_3_): δ 8.59–8.40 (m, 2H), 7.85 (d, *J* = 8.3 Hz, 2H), 7.75 (s, 1H), 7.67 (d, *J* = 8.3 Hz, 2H), 7.18–6.95 (m, 2H), 6.92 (d, *J* = 8.0 Hz, 1H), 3.92 (s, 3H), 2.42 (q, *J* = 7.5 Hz, 2H), 1.24 (t, *J* = 7.5 Hz, 3H) ppm.

^13^C NMR (75 MHz, CDCl_3_): δ 172.58, 164.89, 148.33, 141.50, 130.48, 128.29 (2C), 127.85, 124.06, 121.30, 119.99 (2C), 119.42, 110.09, 55.98, 30.93, 9.69 ppm.

HRMS (pos. ESI-TOF): *m*/*z* calcd for C_17_H_18_N_2_O_3_ 321.1210 [M + Na]^+^; found, 321.1223 [M + Na]^+^.

##### 4-Acetamido-*N*-(2-methoxyphenyl)benzamide (**3k**)

4-Acetamido-*N*-(2-methoxyphenyl)benzamide was synthesized according to general procedure B using 4-amino-*N*-(2-methoxyphenyl)benzamide (100.0 mg, 0.41 mmol), TEA (69.0 μL, 0.49 mmol), acetyl chloride (0.49 mmol, 35 μL) and 3 mL of DCM. The crude was purified by flash chromatography (SiO_2_, cHex/EtOAc 1:0 to 0:1) to obtain compound **3k** in 54% yield (63 mg, 0.22 mmol).

^1^H NMR (300 MHz, DMSO-*d*_6_): δ 10.23 (s, 1H), 9.28 (s, 1H), 7.96–7.85 (m, 2H), 7.79 (dd, *J* = 7.9, 1.6 Hz, 1H), 7.74–7.63 (m, 2H), 7.21–7.12 (m, 1H), 7.08 (dd, *J* = 8.3, 1.5 Hz, 1H), 6.99–6.93 (m, 1H), 3.84 (s, 3H), 2.09 (s, 3H) ppm.

^13^C NMR (75 MHz, DMSO-*d*_6_): δ 169.30, 164.83, 151.75, 142.78, 129.06, 128.87, 127.41, 125.95, 124.50, 120.67, 118.69, 111.77, 56.17, 24.61 ppm.

HRMS (pos. ESI-TOF): *m*/*z* calcd for C_16_H_17_N_2_O_3_ 285.1234 [M + H]^+^; found, 285.1249 [M + H]^+^.

##### 4-Amino-*N*-(2-methoxyphenyl)benzamide (**3l**)

To a cooled suspension of *N*-(2-methoxyphenyl)-4-nitrobenzamide and Pd/C (5%) in methanol, triethylsilane was added. The mixture was allowed to warm up to room temperature and then stirred for 18 h. The mixture was filter under celite and a flash chromatography (SiO_2_, cyclohexane/EtOAc 1:0 to 100% EtOac) was performed. 883 mg (49% yield) of compound **3l** were obtained.

^1^H NMR (300 MHz, DMSO-*d*_6_): δ 8.93 (s, 1H), 7.90 (t, *J* = 7.1 Hz, 1H), 7.70 (d, *J* = 8.2 Hz, 2H), 7.19–7.01 (m, 2H), 6.93 (t, *J* = 7.5 Hz, 1H), 6.67 (d, *J* = 8.2 Hz, 2H), 6.28 (br s, 2H), 3.84 (s, 3H) ppm. Characterization was consistent with the previously published values.^2^

##### *N*-(2-Methoxyphenyl)benzamide (**3m**)

*N*-(2-Methoxyphenyl)benzamide was synthesized according to general procedure B using *o*-methoxyaniline (109.0 mg, 0.81 mmol), TEA (135.0 μL, 0.97 mmol), benzoyl chloride (112 μL, 0.97 mmol) and 3 mL of DCM. The crude was purified by flash chromatography (SiO_2_, cyclohexane/EtOAc 1:0 to 1:1) to obtain compound **3m** as a clear oil in quantitative yield.

^1^H NMR (300 MHz, CDCl_3_): δ 8.58 (t, *J* = 6.1 Hz, 2H), 7.93 (d, *J* = 7.3 Hz, 2H), 7.55 (dd, *J* = 10.5, 6.9 Hz, 3H), 7.09 (dt, *J* = 19.9, 7.5 Hz, 2H), 6.95 (d, *J* = 7.9 Hz, 1H), 3.95 (s, 4H) ppm. The NMR spectrum was consistent with the previously published values.^61^

##### 4-Butyramido-*N*-(quinolin-8-yl)benzamide (**4a**)

4-Butyramido-*N*-(quinolin-8-yl)benzamide was synthesized according to general procedure B using 4-amino-*N*-(quinolin-8-yl)benzamide (100.0 mg, 0.38 mmol), TEA (63.0 μL, 0.46 mmol), butyryl chloride (44 μL, 0.46 mmol) and 6 mL of DCM. The crude product was concentrated in vacuum, redissolved in hot DCM and filtrated through short plug of silica. The filtrate was slowly cooled to 0 °C, and precipitated crystals were collected using a Buchner funnel to obtain compound **4a** in 61% yield (77 mg, 0.23 mmol, white solid).

^1^H NMR (300 MHz, DMSO-*d*_6_): δ 10.60 (s, 1H), 10.25 (s, 1H), 8.98 (dd, *J* = 5.0, 2.8 Hz, 1H), 8.73 (dd, *J* = 7.6, 1.5 Hz, 1H), 8.44 (d, *J* = 7.9 Hz, 1H), 8.05–7.95 (m, 2H), 7.83 (d, *J* = 8.4 Hz, 2H), 7.76–7.61 (m, 3H), 2.35 (t, *J* = 7.3 Hz, 2H), 1.64 (h, *J* = 7.4 Hz, 2H), 0.93 (t, *J* = 7.3 Hz, 2H) ppm.

^13^C NMR (75 MHz, DMSO-*d*_6_): δ 173.04, 164.43, 149.61, 143.32, 138.69, 137.24, 134.60, 128.80, 128.52, 128.30, 127.54, 122.81, 122.56, 119.16, 116.86, 30.08, 9.97 ppm.

HRMS (pos. ESI-TOF): *m*/*z* calcd for C_20_H_19_N_3_O_2_ 334.1550 [M + H]^+^; found, 334.1544 [M + H]^+^.

##### 4-Propionamido-*N*-(quinolin-8-yl)benzamide (**4b**)

4-Propionamido-*N*-(quinolin-8-yl)benzamide was synthesized according to general procedure B using 4-amino-*N*-(quinolin-8-yl)benzamide (100.0 mg, 0.38 mmol), TEA (63.0 μL, 0.46 mmol), propionyl chloride (40.0 μL, 0.46 mmol) and 6 mL of DCM. The crude product was concentrated in vacuum, redissolved in hot DCM and filtrated through short plug of silica. The filtrate was slowly cooled to 0 °C, and precipitated crystals were collected using a Buchner funnel to obtain compound **4b** in 73% yield (89 mg, 0.23 mmol, white solid).

^1^H NMR (300 MHz, DMSO-*d*_6_): δ 10.59 (s, 1H), 10.24 (s, 1H), 8.97 (dd, *J* = 4.3, 1.7 Hz, 1H), 8.72 (dd, *J* = 7.5, 1.5 Hz, 1H), 8.44 (dd, *J* = 8.3, 1.7 Hz, 1H), 8.05–7.95 (m, 2H), 7.87–7.78 (m, 2H), 7.76–7.58 (m, 3H), 2.38 (q, *J* = 7.5 Hz, 2H), 1.10 (t, *J* = 7.5 Hz, 3H) ppm.

^13^C NMR (75 MHz, DMSO-*d*_6_): δ 173.04, 164.43, 149.61, 143.32, 138.69, 137.24, 134.60, 128.80, 128.52, 128.30, 127.54, 122.81, 122.56, 119.16, 116.86, 30.08, 9.97 ppm.

HRMS (pos. ESI-TOF): *m*/*z* calcd for C_19_H_17_N_3_O_2_ 320.1399 [M + H]^+^; found, 320.1408 [M + H]^+^.

##### 4-Acetamido-*N*-(quinolin-8-yl)benzamide (**4c**)

4-Acetamido-*N*-(quinolin-8-yl)benzamide was synthesized according to general procedure B using 4-amino-*N*-(quinolin-8-yl)benzamide (100.0 mg, 0.38 mmol), TEA (63.0 μL, 0.46 mmol), acetyl chloride (32 μL, 0.46 mmol) and 6 mL of DCM. The crude product was concentrated in vacuum, redissolved in hot DCM and filtrated through short plug of silica. The filtrate was slowly cooled to 0 °C, and precipitated crystals were collected using a Buchner funnel to obtain compound **4c** in 95% yield (110 mg, 0.36 mmol, white solid).

^1^H NMR (300 MHz, CDCl_3_): δ 10.73 (s, 1H), 8.96–8.80 (m, 2H), 8.21 (dd, *J* = 8.3, 1.6 Hz, 1H), 8.11–8.03 (m, 2H), 7.71 (d, *J* = 8.3 Hz, 2H), 7.66–7.38 (m, 4H), 2.24 (s, 3H) ppm. Characterization was consistent with the previously published values.^62^

##### 4-Amino-*N*-(quinolin-8-yl)benzamide (**4d**)

4-Amino-*N*-(quinolin-8-yl)benzamide was synthesized according to general procedure A using 8-aminoquinolinone (0.5 g, 3.5 mmol), 4-*tert*-butoxycarbonylbenzoic acid (0.82 g, 3.5 mmol), DMAP (48.9 mg, 0.35 mmol), and EDCI (1.08 mg, 7.0 mmol). After completion of the coupling reaction, the crude mixture was worked up and submitted to the deprotection step without further purification. The crude product was successfully deprotected using 34 mL DCM/TFA (4:1) solution. The crude was purified by flash chromatography (SiO_2_, cyclohexane/EtOAc 1:1 to 100% EtOAc) to obtain compound **4d** in 38% yield in 2 steps (350 mg, 0.24 mmol, white powder).

^1^H NMR (300 MHz, DMSO-*d*_6_): δ 10.45 (s, 1H), 8.97 (dd, *J* = 4.3, 1.7 Hz, 1H), 8.73 (dd, *J* = 7.3, 1.7 Hz, 1H), 8.44 (dd, *J* = 8.3, 1.7 Hz, 1H), 7.79–7.71 (m, 2H), 7.71–7.48 (m, 3H), 6.71 (d, *J* = 8.6 Hz, 2H) ppm. ^13^C NMR (75 MHz, CDCl_3_): δ 164.30, 152.35, 148.90, 138.00, 136.81, 134.53, 128.70 (2C), 127.81, 127.14, 122.23, 121.29, 120.70, 115.75, 113.25 (2C) ppm. HRMS (pos. ESI-TOF): *m*/*z* calcd for C_16_H_13_N_3_O 264.1136 [M + H]^+^, 264.1140 found [M + H]^+^.

##### *N*-(Quinolin-8-yl)benzamide (**4e**)

*N*-(Quinolin-8-yl)benzamide was synthesized according to general procedure B using *N*-(quinolin-8-yl)benzamide (100.0 mg, 0.69 mmol), TEA (115.0 μL, 0.97 mmol), benzoyl chloride (95 μL, 0.83 mmol) and 3 mL of DCM. The crude product was concentrated in vacuum, redissolved in hot DCM and filtrated through short plug of silica. The filtrate was slowly cooled to 0 °C, and precipitated crystals were collected using a Buchner funnel in quantitative yield.

^1^H NMR (300 MHz, CDCl_3_): δ 10.79 (s, 1H), 8.97 (dd, *J* = 7.4, 1.6 Hz, 1H), 8.88 (dd, *J* = 4.3, 1.7 Hz, 1H), 8.22 (dd, *J* = 8.3, 1.7 Hz, 1H), 8.17–8.09 (m, 2H), 7.68–7.56 (m, 4H), 7.56 (dd, *J* = 5.3, 3.5 Hz, 1H), 7.51 (dd, *J* = 8.3, 4.3 Hz, 1H) ppm. The NMR spectrum was consistent with the previously published values.^63^

##### *N*-(2-Methoxyphenyl)-4-(3-(4-oxo-3,4-dihydroquinazolin-2-yl)propanamido)benzamide (**1**)

4-Amino-*N*-(2-methoxyphenyl)benzamide (133 mg, 0.55 mmol, 1.2 equiv) was added to a suspension of 3-(4-oxo-3,4-dihydroquinazolin-2-yl)propanoic acid (100 mg, 0.46 mmol, 1.0 equiv) and HATU (93 mg, 0.55 mmol, 1.2 equiv) in anhydrous DMF (4.6 mL). The mixture was stirred at room temperature for 48 h. It was then diluted with DCM (10 mL) and quenched with water (5 mL). The phases were separated and the aqueous phase was extracted twice with DCM (10 mL). Combined organic phases were dried over MgSO_4_, filtered and concentrated under vacuum. The crude product was further purified by column chromatography to obtain compound **1** (8 mg, 4% yield) as an amorphous solid.

^1^H NMR (300 MHz, DMSO-*d*_6_): δ 12.28 (br s, 1H), 10.38 (br s, 1H), 9.29 (s, 1H), 8.10 (dd, *J* = 8.0, 1.5 Hz, 1H), 7.95–7.88 (m, 2H), 7.83–7.71 (m, 4H), 7.56 (d, *J* = 8.2 Hz, 1H), 7.51–7.43 (m, 1H), 7.16 (dd, *J* = 7.3, 1.7 Hz, 1H), 7.09 (dd, *J* = 8.3, 1.5 Hz, 1H), 6.96 (td, *J* = 7.5, 1.5 Hz, 1H), 3.84 (s, 3H), 3.02–2.88 (m, 4H) ppm.

Characterization was consistent with the previously published values.^64^

##### 4-(3-(4-Oxo-3,4-dihydroquinazolin-2-yl)propanamido)-*N*-(quinolin-8-yl)benzamide (**2**)

4-Amino-*N*-(quinolin-8-yl)benzamide (47 mg, 0.18 mmol, 1.0 equiv) was added to a suspension of 3-(4-oxo-3,4-dihydroquinazolin-2-yl)propanoic acid (58 mg, 0.46 mmol, 1.5 equiv) and HATU (39 mg, 0.23 mmol, 1.3 equiv) in anhydrous DMF (1.8 mL). The mixture was stirred at room temperature for 48 h. It was then diluted with DCM (10 mL) and quenched with water (5 mL). The phases were separated and the aqueous phase was extracted twice with DCM (10 mL). Combined organic phases were dried over MgSO_4_, filtered and concentrated under vacuum. The crude product was further purified by column chromatography to obtain compound **2** (6 mg, 7% yield) as an amorphous solid.

^1^H NMR (300 MHz, DMSO-*d*_6_): δ 12.28 (br s, 1H), 10.60 (br s, 1H), 10.47 (br s, 1H), 8.97 (dd, *J* = 4.3, 1.7 Hz, 1H), 8.72 (dd, *J* = 7.5, 1.4 Hz, 1H), 8.46 (dd, *J* = 8.4, 1.6 Hz, 1H), 8.09 (dd, *J* = 7.9, 1.6 Hz, 1H), 8.00 (d, *J* = 8.6 Hz, 2H), 7.86–7.63 (m, 6H), 7.56 (d, *J* = 8.1 Hz, 1H), 7.50–7.42 (m, 1H), 3.05–2.92 (m, 4H) ppm. Characterization was consistent with the previously published values.^59^

##### *N*-(2-Methoxyphenyl)-4-(3-methyl-3-(2-(4-oxo-3,4-dihydroquinazolin-2-yl)ethyl)ureido)benzamide (**5**)

2-(2-Aminoethyl)-4(3*H*)-quinazolinone dihydrochloride hydrate (100 mg, 0.357 mmol) was dissolved in MeOH (2 mL) at room temperature, followed by the addition of formaldehyde (37% in water, 1.1 equiv, 30 μL, 0.400 mmol) and potassium acetate (3 equiv, 105 mg, 1.07 mmol). After 1 h, sodium cyanoborohydride (3 equiv, 67 mg, 1.07 mmol) was added to the stirring solution. After 3 h, the reaction was quenched with a saturated solution of Na_2_CO_3_ in water and the crude was extracted three times with DCM (50 mL). The organic phases were reunited, dried over Na_2_SO_4_ and concentrated in vacuum. The crude was dissolved in acetonitrile and water (1:1) mixture and then a purification by reversed-phase preparative HPLC (5 to 95% acetonitrile in water with 0.1% TFA) was carried out. However, the product contained still some impurities. The crude was dissolved in dry DCM (1 mL) at room temperature, followed by the addition of triphosgene (3 equiv, 184 mg, 0.619 mmol) and cesium carbonate (3 equiv, 201 mg, 0.619 mmol). After 3 h, the reaction was concentrated in vacuum and the crude was dissolved in 1 mL of ACN followed by the addition of 4-amino-*N*-(2-methoxyphenyl)benzamide (82 mg, 0.206 mmol). After 3 h at room temperature, 50 mL of a saturated solution of Na_2_CO_3_ in water was added to the crude and it was extracted three times with DCM (50 mL). The organic phases were reunited, dried over Na_2_SO_4_ and concentrated in vacuum. The crude was dissolved in acetonitrile and water (1:1) mixture and then purified by reversed-phase preparative HPLC (5 to 95% acetonitrile in water with 0.1% TFA) to obtain compound **5** (7 mg, 3% yield after two steps) as an amorphous solid.

^1^H NMR (600 MHz, DMSO-*d*_6_): δ 9.16 (s, 1H), 8.62 (s, 1H), 8.11 (d, *J* = 8.0 Hz, 1H), 7.85–7.73 (m, 4H), 7.66–7.60 (m, 1H), 7.54–7.41 (m, 3H), 7.17–7.12 (m, 1H), 7.08 (d, *J* = 8.2 Hz, 1H), 6.95 (t, *J* = 7.6 Hz, 1H), 3.84 (s, 3H), 3.79 (t, *J* = 7.0 Hz, 3H), 3.00 (s, 3H), 2.94 (dd, *J* = 12.2, 5.4 Hz, 2H) ppm.

^13^C NMR (151 MHz DMSO-*d*_6_): δ 175.91, 172.69, 164.75, 159.04, 158.81, 156.62, 151.70, 149.73, 142.61, 135.56, 129.28, 128.87, 128.25, 127.46, 126.93, 125.91, 124.40, 120.68, 118.97, 111.81, 56.21, 42.19, 33.66, 33.33 ppm.

HRMS (pos. ESI-TOF): *m*/*z* calcd for C_26_H_25_N_5_O_4_ 472.1985 [M + H]^+^; found, 472.1970 [M + H]^+^.

##### 2-(2-((Thiophen-2-ylmethyl)amino)ethyl)quinazolin-4(3*H*)-one

2-(2-Aminoethyl)-4(3*H*)-quinazolinone dihydrochloride hydrate (150 mg, 0.535 mmol) was dissolved in MeOH (2 mL) at room temperature, followed by the addition of thiophene-2-carbaldehyde (1.1 equiv, 66 mg, 0.589 mmol) and potassium acetate (3 equiv, 340 mg, 1.61 mmol). After 1 h, sodium triacetoxyborohydride (3 equiv, 158 mg, 1.61 mmol) was added to the stirring solution. After 3 h, the reaction was quenched with a saturated solution of Na_2_CO_3_ in water and the crude was extracted three times with DCM (50 mL). The organic phases were reunited, dried over Na_2_SO_4_ and concentrated in vacuum. The crude was dissolved in acetonitrile and water (1:1) mixture and then purified by reversed-phase preparative HPLC (5 to 95% acetonitrile in water with 0.1% TFA) to obtain the title compound (82 mg, 38% yield) as an amorphous solid.

^1^H NMR (300 MHz, CDCl_3_): δ 8.24 (d, *J* = 7.8 Hz, 1H), 7.71 (t, *J* = 7.5 Hz, 1H), 7.61 (d, *J* = 8.1 Hz, 1H), 7.42 (t, *J* = 7.4 Hz, 1H), 7.23 (d, *J* = 4.9 Hz, 1H), 7.04–6.87 (m, 2H), 4.12 (s, 2H), 3.15 (s, 2H), 2.90 (s, 2H) ppm.

^13^C NMR (300 MHz, CDCl_3_): δ 162.31, 156.05, 148.77, 134.36, 126.90, 126.78, 126.57, 126.36, 126.27, 126.06, 125.20, 121.34, 47.48, 45.11, 33.61 ppm. (Given the low intensity of the TFA carbons, they were not detected in the ^13^C NMR)

##### *N*-(2-Methoxyphenyl)-4-(3-(2-(4-oxo-3,4-dihydroquinazolin-2-yl)ethyl)-3-(thiophen-2-ylmethyl)ureido)benzamide (**6**)

2-(4-oxo-3,4-dihydroquinazolin-2-yl)-*N*-(thiophen-2-ylmethyl)ethan-1-aminium 2,2,2-trifluoroacetate (50 mg, 0.206 mmol) was dissolved in dry DCM (1 mL) at room temperature, followed by the addition of triphosgene (3 equiv, 184 mg, 0.619 mmol) and cesium carbonate (3 equiv, 201 mg, 0.619 mmol). After 3 h, the reaction was concentrated in vacuum and the crude was dissolved in 1 mL of ACN followed by the addition of 4-amino-*N*-(2-methoxyphenyl)benzamide (82 mg, 0.206 mmol). After 3 h at room temperature, 50 mL of a saturated solution of Na_2_CO_3_ in water was added to the crude and it was extracted three times with DCM (50 mL). The organic phases were reunited, dried over Na_2_SO_4_ and concentrated in vacuum. The crude was dissolved in acetonitrile and water (1:1) mixture and then purified by reversed-phase preparative HPLC (5 to 95% acetonitrile in water with 0.1% TFA) to obtain compound **6** (15 mg, 13% yield) as an amorphous solid.

^1^H NMR (600 MHz, CDCl_3_): δ 8.46 (s, 1H), 8.36 (dd, *J* = 8.1, 1.6 Hz, 1H), 8.15 (dd, *J* = 8.0, 1.4 Hz, 1H), 7.92 (d, *J* = 8.3 Hz, 1H), 7.85–7.76 (m, 2H), 7.65 (d, *J* = 8.3 Hz, 2H), 7.57 (t, *J* = 7.7 Hz, 1H), 7.32 (d, *J* = 8.2 Hz, 2H), 7.24 (dd, *J* = 5.1, 1.1 Hz, 1H), 7.11–7.04 (m, 2H), 6.99–6.93 (m, 2H), 6.89 (dd, *J* = 8.2, 1.3 Hz, 1H), 4.91 (s, 2H), 4.05 (t, *J* = 6.0 Hz, 2H), 3.90 (s, 3H), 3.62 (t, *J* = 6.2 Hz, 2H) ppm.

^13^C NMR (151 MHz DMSO-*d*_6_): δ 164.41, 161.31, 154.54, 151.14, 143.48, 141.10, 134.68, 127.95, 127.48, 127.07, 126.67, 126.56, 126.37, 125.94, 125.78, 125.29, 125.16, 123.83, 123.74, 120.65, 120.19, 118.98, 118.86, 111.28, 55.73, 44.89, 43.63, 32.92 ppm.

HRMS (pos. ESI-TOF): *m*/*z* calcd for C_30_H_27_N_5_O_4_S 554.1862 [M + H]^+^; found, 554.1849 [M + H]^+^.

##### 4-((Tetrahydro-2*H*-pyran-2-yl)oxy)benzoic Acid

4-Hydroxybenzoic acid (2 g, 14.5 mmol), diethyl ether (20 mL) and *p*-toluenesulfonic acid (pTSA) (0.036 g, 0.256 mmol) were mixed in a flask. The resulting suspension was stirred at room temperature. 3,4-Dihydro-2*H*-pyran (DHP) (3.97 mL, 43.5 mmol) was added to the mixture. The suspension turned clear soon after the addition of DHP and a white crystalline precipitate formed. The mixture was then stirred at room temperature for 18 h. The resulting precipitates were collected by vacuum filtration and washed with ethyl ether. White crystals were recovered as the product (1.12 g, 35% yield).

^1^H NMR (300 MHz, CDCl_3_): δ 8.14–8.00 (m, 2H), 7.18–7.07 (m, 2H), 5.56 (t, *J* = 3.2 Hz, 1H), 3.89 (ddd, *J* = 11.2, 9.6, 3.1 Hz, 1H), 3.66 (ddt, *J* = 11.4, 4.2, 2.4 Hz, 1H), 2.15–1.86 (m, 3H), 1.84–1.56 (m, 2H) ppm.

##### 4-Hydroxy-*N*-(2-methoxyphenyl)benzamide

DIPEA (2.20 mL, 12.6 mmol) and HATU (1.92 g, 5.05 mmol) were added to a solution of 5-cyclopropyl-6-(4-fluorobenzyl)picolinic acid (1.12 g, 5.05 mmol) in DMF (10 mL) at room temperature. The mixture was stirred at room temperature for 30 min and then *o*-anidisine (0.570 mL, 5.05 mmol) was added. The mixture was stirred at room temperature for 18 h. Solvent was evaporated and the mixture solubilized in DCM and washed successively with a saturated solution of sodium bicarbonate, 1 M HCl and brine. 1.25 g (75% yield) of *N*-(2-methoxyphenyl)-4-((tetrahydro-2*H*-pyran-2-yl)oxy)benzamide were obtained and used in the next step without further purification.

To a solution of *N*-(2-methoxyphenyl)-4-((tetrahydro-2*H*-pyran-2-yl)oxy)benzamide (1.25 g, 3.81 mmol) in methanol (10 mL) at room temperature was added pTSA (0.072 g, 0.38 mmol). The mixture was stirred at room temperature for 1 h. The crude was purified by flash chromatography (SiO_2_, cyclohexane/EtOAc 1:1) to provide 769 mg (83%) of the title compound.

^1^H NMR (300 MHz, (CD_3_)_2_CO): δ 9.11 (s, 1H), 8.74 (s, 1H), 8.45–8.39 (m, 1H), 7.94–7.85 (m, 2H), 7.14–7.03 (m, 2H), 7.02–6.93 (m, 3H), 3.95 (s, 3H) ppm.

^13^C NMR (75 MHz, (CD_3_)_2_CO): δ 164.25, 160.68, 148.92, 148.85, 129.15, 128.26, 126.34, 123.66, 120.52, 120.12, 120.01, 115.29, 110.39, 55.46, 37.88 ppm.

HRMS (pos. ESI-TOF): *m*/*z* calcd for C_14_H_13_NO_3_ 266.0788 [M + Na]^+^; found, 266.0782 [M + H]^+^.

##### Methyl 3-((*trans*-4-Oxo-3,4-dihydroquinazolin-2-yl)amino)cyclobutane-1-carboxylate

*trans*-Methyl 3-amino cyclobutane-1-carboxylatehydrochloride (118 mg, 0.91 mmol) and triethylamine (0.174 mL, 1.25 mmol) were added to a stirring solution of 2-chloro-3,4-dihydroquinazolin-4-one (150 mg, 0.83 mmol) in ethanol (10 mL). The mixture was heated to 80 °C and stirred. The crude was purified by flash chromatography (SiO_2_, 100% cyclohexane to 100% EtOAc) to provide 126 mg (55%) of the title compound.

^1^H NMR (300 MHz, DMSO-*d*_6_): δ 10.85 (s, 1H), 7.89 (dd, *J* = 8.0, 1.6 Hz, 1H), 7.67–7.49 (m, 1H), 7.27 (d, *J* = 8.2 Hz, 1H), 7.18–7.03 (m, 1H), 6.64 (s, 1H), 4.70–4.43 (m, 1H), 3.67 (s, 3H), 3.08 (tdd, *J* = 9.6, 4.0, 2.2 Hz, 1H), 2.67–2.41 (m, 2H), 2.28 (ddd, *J* = 12.8, 10.1, 7.9 Hz, 2H) ppm.

^13^C NMR (75 MHz, DMSO-*d*_6_): δ 176.03, 162.64, 151.33, 150.00, 148.32, 135.47, 134.63, 127.64, 126.93, 126.74, 126.39, 125.14, 122.34, 121.23, 117.99, 52.15, 43.99, 33.46, 32.42 ppm.

HRMS (pos. ESI-TOF): *m*/*z* calcd for C_14_H_15_N_3_O_3_ 274.1192 [M + H]^+^; found, 274.1183 [M + H]^+^.

##### *N*-(2-Methoxyphenyl)-4-(3-((*trans*-4-oxo-3,4-dihydroquinazolin-2-yl)amino)cyclobutane-1-carboxamido)benzamide (**7**)

1 M NaOH (1.24 mL, 124 mmol) was added to a stirring solution of methyl 3-((4-oxo-3,4-dihydroquinazolin-2-yl)amino)cyclobutane-1-carboxylate (113 mg,0.42 mmol) in methanol (5 mL). After 24 h, the mixture was adjusted to pH 2 with 1 M HCl and then extracted with ethyl acetate. The organic phase was washed with brine, dried over Na_2_SO_4_ and concentrated in vacuum.

DIPEA (104 μL, 0.60 mmol) and HATU (181 mg, 0.48 mmol) were then added to a solution of 3-((4-oxo-3,4-dihydroquinazolin-2-yl)amino)cyclobutane-1-carboxylic acid (67.8 mg, 0.26 mmol) in DMF (5 mL) at room temperature. The mixture was stirred at room temperature for 30 min and then 4-amino-*N*-(2-methoxyphenyl)benzamide (57.6 mg, 0.24 mmol) was added. The mixture was stirred at room temperature for 18 h. The crude residues were dissolved in acetonitrile and water (1:1) mixture and then purified by reversed-phase preparative HPLC (5 to 40% acetonitrile in water with 0.1% TFA).

^1^H NMR (300 MHz, DMSO-*d*_6_): δ 10.25 (s, 1H), 9.31 (s, 1H), 7.96 (d, *J* = 8.5 Hz, 3H), 7.80 (d, *J* = 7.2 Hz, 3H), 7.72 (s, 1H), 7.45 (d, *J* = 8.2 Hz, 1H), 7.30 (d, *J* = 7.6 Hz, 1H), 7.18 (t, *J* = 7.8 Hz, 1H), 7.25–7.06 (m, 2H), 6.97 (t, *J* = 7.6 Hz, 1H), 4.62 (s, 1H), 3.85 (s, 3H), 2.68 (s, 2H), 2.41 (t, *J* = 9.4 Hz, 2H) ppm.

^13^C NMR (75 MHz, DMSO-*d*_6_): δ 164.76, 151.77, 142.81, 129.13, 128.91, 127.41, 125.98, 124.55, 120.68, 118.85, 111.79, 56.19, 34.57, 32.99 ppm.

HRMS (pos. ESI-TOF) *m*/*z* calcd for C_27_H_25_N_5_O_4_ 484.1985 [M + H]^+^; found, 484.1978 [M + H]^+^.

##### Methyl (*cis*)-3-((4-Oxo-3,4-dihydroquinazolin-2-yl)amino)cyclobutane-1-carboxylate

*cis*-Methyl 3-amino cyclobutane-1-carboxylate hydrochloride (1.2 equiv, 165 mg, 1.00 mmol) and DIPEA (2.5 equiv, 0.37 mL, 2.08 mmol) were added to a stirring solution of 2-chloro-3,4-dihydroquinazolin-4-one (150 mg, 0.83 mmol) in DMSO (2 mL). After 6 h at 120 °C, 2 mL of solution acetonitrile and water (1:1) were added to the mixture and the crude was purified by reversed-phase preparative HPLC (5 to 95% acetonitrile in water with 0.1% TFA) to obtain the title compound (253 mg, 79% yield) as an amorphous solid.

^1^H NMR (MHz, DMSO-*d*_6_): δ 7.94 (dd, *J* = 7.9, 1.5 Hz, 1H), 7.74–7.65 (m, 1H), 7.40 (d, *J* = 8.2 Hz, 1H), 7.27 (t, *J* = 7.5 Hz, 1H), 4.46–4.27 (m, 1H), 3.62 (s, 3H), 3.00–2.82 (m, 1H), 2.69–2.51 (m, 2H), 2.31–2.13 (m, 2H) ppm. ^13^C NMR (75 MHz, DMSO-*d*_6_): δ 174.61, 161.59, 149.73, 135.54, 132.45, 126.89, 123.96, 120.85, 117.03, 52.09, 42.10, 33.81, 30.82 ppm.

##### *N*-(2-Methoxyphenyl)-4-(*cis*-3-((4-oxo-3,4-dihydroquinazolin-2-yl)amino)cyclobutane-1-carboxamido)benzamide (**8**)

Lithium hydroxide monohydrate (4 equiv, 95 mg, 2.27 mmol) was added to a stirring solution of *N*-(*Cis*-3-(methoxycarbonyl)cyclobutyl)-4-oxo-3,4-dihydroquinazolin-2-aminium 2,2,2-trifluoroacetate (220 mg, 0.568 mmol) in 3 mL of a solvent mixture Water/THF (1:1). After 1 h at room temperature, the mixture was adjusted to pH 2 with 1 M HCl and then extracted three times with DCM. The organic phases were reunited, dried over Na_2_SO_4_ and concentrated in vacuum. The crude was first dissolved in DMF (3 mL) at room temperature, then DIPEA (2 equiv, 0.20 mL, 1.13 mmol) and HATU (1 equiv, 215 mg, 0.567 mmol) were added to the solution. The mixture was stirred at room temperature for 15 min and then 4-amino-*N*-(2-methoxyphenyl)benzamide (1 equiv, 137 mg, 0.057 mmol) was added. The mixture was stirred at room temperature for 18 h. The DMF was evaporated and the crude was dissolved in acetonitrile and water (1:1) mixture and then purified by reversed-phase preparative HPLC (5 to 95% acetonitrile in water with 0.1% TFA). The pure product was then extracted three times from a water solution of Na_2_CO_3_ with DCM to afford compound **8** (145 mg, 53%) as an amorphous solid.

^1^H NMR (600 MHz, DMSO-*d*_6_): δ 10.21 (s, 1H), 9.27 (s, 1H), 7.96 (dd, *J* = 7.9, 1.6 Hz, 1H), 7.95–7.92 (m, 2H), 7.80 (dd, *J* = 7.9, 1.7 Hz, 1H), 7.78–7.74 (m, 2H), 7.73–7.69 (m, 1H), 7.42 (d, *J* = 8.2 Hz, 1H), 7.30–7.25 (m, 1H), 7.16 (td, *J* = 7.8, 1.7 Hz, 1H), 7.09 (dd, *J* = 8.2, 1.3 Hz, 1H), 6.96 (td, *J* = 7.6, 1.4 Hz, 1H), 4.45–4.37 (m, 1H), 3.84 (s, 3H), 3.04–2.98 (m, 1H), 2.67–2.60 (m, 2H), 2.33–2.25 (m, 2H) ppm.

^13^C NMR (151 MHz, DMSO-*d*_6_): δ 175.91, 172.69, 164.75, 159.04, 158.81, 156.62, 151.70, 149.73, 142.61, 135.56, 129.28, 128.87, 128.25, 127.46, 126.93, 125.91, 124.40, 120.68, 118.97, 111.81, 56.21, 42.19, 33.66, 33.33 ppm.

HRMS (pos. ESI-TOF): *m*/*z* calcd for C_27_H_26_N_5_O_4_S 484.1985 [M + H]^+^; found, 484.1976 [M + H]^+^.

##### *tert*-Butyl (*R*)-3-(4-((2-Methoxyphenyl)carbamoyl)phenoxy)pyrrolidine-1-carboxylate

A solution of 4-hydroxy-*N*-(2-methoxyphenyl)benzamide (110 mg, 0.45 mmol) and K_2_CO_3_ (104 mg, 0.75 mmol) in THF (5 mL) was stirred for 30 min. Then *tert*-butyl-(*R*)-3-((methylsulfonyl)oxy)pyrrolidine-1-carboxilate was added and the mixture heated until reflux. After 4 h was observed by LC–MS the completion of the reaction. The base was filtered and the crude was purified by flash chromatography (SiO_2_, cyclohexane 100% to cyclohexane/EtOAc 1:1) to provide 109 mg (70%) of the title compound.

^1^H NMR (300 MHz, CDCl_3_): δ 8.57–8.48 (m, 2H), 7.84 (td, *J* = 8.5, 7.7, 0.0 Hz, 2H), 7.15–6.89 (m, 4H), 4.98 (tt, *J* = 4.4, 2.2 Hz, 1H), 3.95 (s, 3H), 3.74–3.43 (m, 4H), 2.98 (s, 1H), 2.32–2.06 (m, 2H), 1.49 (s, 9H) ppm.

^13^C NMR (75 MHz, CDCl_3_): δ 164.70, 162.61, 159.99, 154.52, 148.09, 129.04, 127.94, 127.90, 125.83, 123.70, 121.23, 119.78, 115.62, 115.31, 109.92, 79.65, 55.84, 51.50, 43.90, 36.57, 31.50, 30.33, 28.52 ppm.

HRMS (pos. ESI-TOF): *m*/*z* calcd for C_23_H_28_N_2_O_5_ 413.2076 [M + H]^+^; found, 413.2073 [M + H]^+^.

##### (*R*)-*N*-(2-Methoxyphenyl)-4-((1-(4-oxo-3,4-dihydroquinazolin-2-yl)pyrrolidin-3-yl)oxy)benzamide (**9**)

TFA (1 mL) was added to a stirring solution of *tert*-butyl (*R*)-3-(4-((2-methoxyphenyl)carbamoyl)phenoxy)pyrrolidine-1-carboxylate (109 mg, 0.26 mmol) in DCM (1 mL). The mixture was stirred at rt for 45 min. The solvent was coevaporated with toluene.

*N*-(2-Methoxyphenyl)-4-(pyrrolidin-3-yloxy)benzamide (66 mg, 0.21 mmol) and triethylamine (0.040 mL, 0.29 mmol) were added to a stirring solution of 2-chloro-3,4-dihydroquinazolin-4-one (35 mg, 0.19 mmol) in ethanol (5 mL). The mixture was heated to 80 °C and stirred. The resulting precipitates were collected by vacuum filtration and washed with methanol. White crystals were recovered as the product (35 mg, 40% yield).

^1^H NMR (300 MHz, DMSO-*d*_6_): δ 11.27 (s, 1H), 9.31 (s, 1H), 7.97 (d, *J* = 8.3 Hz, 2H), 7.91 (d, *J* = 7.7 Hz, 1H), 7.79 (d, *J* = 7.9 Hz, 1H), 7.57 (t, *J* = 7.7 Hz, 1H), 7.30–7.18 (m, 1H), 7.13 (dt, *J* = 14.9, 7.7 Hz, 5H), 6.98 (d, *J* = 7.8 Hz, 1H), 5.28 (s, 1H), 3.85 (s, 5H), 3.63 (d, *J* = 9.6 Hz, 1H), 2.28 (s, 2H) ppm.

^13^C NMR (75 MHz, DMSO-*d*_6_): δ 164.77, 163.36, 159.97, 151.84, 151.50, 149.72, 134.79, 129.97, 127.46, 126.43, 125.96, 124.94, 124.66, 121.89, 120.66, 117.11, 115.61, 111.79, 76.21, 56.51, 56.18, 52.78, 45.24, 30.85 ppm.

HRMS (pos. ESI-TOF): *m*/*z* calcd for C_26_H_24_N_4_O_4_ 457.1870 [M + H]^+^; found, 457.1876 [M + H]^+^.

##### (*S*)-*N*-(2-Methoxyphenyl)-4-((1-(4-oxo-3,4-dihydroquinazolin-2-yl)pyrrolidin-3-yl)oxy)benzamide (**10**)

The procedure employed for compound **9** was also followed to obtain compound **10**, employing *tert*-butyl-(*S*)-3-((methylsulfonyl)oxy)pyrrolidine-1-carboxilate as starting material.

## Data Availability

The coordinates and structural factors of the crystal structures were deposited in the PDB and are accessible with IDs 7OJO and 8B6M. The raw diffraction data are available at Fairdata (10.23729/f07e6695-a268-4c7f-8a43-b48778f0a4a5).

## Supporting Information Available

Table S1 contains the binding parameters of fragments of TNKS2 ligand S3. Table S2 holds the calculated Δ*G* contributions of linkers for ligand 3. Table S3 contains key characteristics of the molecules included in the computational assessment of the linker replacement studies. Table S4 contains the calculated LLE data for all fragments. Table S5 contains the data collection and refinement statistics of the obtained X-ray structures. Experimental procedures for the synthesis of all fragments of the adenosine site binder S3. HPLC chromatograms of the final compounds (PDF)
Molecular formula strings for all final compounds (CSV)

## Abbreviations

ACN: acetonitrile
ADE: adenosine-binding pocket
ADP: adenosine diphosphate
ART: ADP-ribosyltransferase
BTP: Bis-Tris propane
DCM: dichloromethane
DHP: 3,4-dihydro-2*H*-pyran
DMAP: *N*,*N*-dimethylpyridin-4-amine
DMF: *N*,*N*-dimethylformamide
DMSO: dimethyl sulfoxide
EDCI: 1-ethyl-3-(3-(dimethylamino)propyl)carbodiimide
ELSD: evaporative light scattering detector
ESI: electrospray ionization
HATU: 1-[bis(dimethylamino)methylene]-1*H*-1,2,3-triazolo[4,5-*b*]pyridinium 3-oxide hexafluorophosphate
HEPES: 4-(2-hydroxyethyl)-1-piperazineethanesulfonic acid
HPLC: high-performance liquid chromatography
HRMS: high-resolution mass spectrometry
LE: ligand efficiency
NAD: nicotinamide adenine dinucleotide
NI: nicotinamide-binding pocket
NMR: nuclear magnetic resonance
PARP: poly(ADP-ribose) polymerase
TCEP: tris(2-carboxyethyl)phosphine
