# Supplementary material for: Deconstruction of Dual-Site Tankyrase Inhibitors Provides Insights into Binding Energetics and Suggests Critical Hotspots for Ligand Optimization
Source: J Med Chem. 2025 Mar 26;68(7):7263–79. doi: 10.1021/acs.jmedchem.4c02845 (PMC11997991; doi:10.1021/acs.jmedchem.4c02845)
Supplement: Supplementary file 1 — jm4c02845_si_001.pdf [file jm4c02845_si_001.pdf]

Supporting Information

Deconstruction of dual-site tankyrase inhibitors provides insights into binding energetics and suggests critical hotspots for ligand optimization

Sven T. Sowa,[#a] Murat Kücükdisli,[#b] Yelena Mostinski,[#b] David A. Schaller,[c] Carolina S. Vinagreiro,[b] Davide Cirillo,[b] Chiara Bosetti,[a] Shoshy Alam Brinch,[de] Kirsten van Laar,[f] Anita Wegert,[f] Ruben G.G. Leenders,[f] Stefan Krauss,[de] Jo Waaler,[de] Andrea Volkamer,[cg] Lari Lehtiö*[a] and Marc Nazaré*[b]*

[a]Faculty for Biochemistry and Molecular Medicine & Biocenter Oulu, University of Oulu, Aapistie 7, 90220 Oulu, Finland.
Corresponding author email address: lari.lehtio@oulu.fi

[b]Medicinal Chemistry, Leibniz-Forschungsinstitut für Molekulare Pharmakologie (FMP), Campus Berlin Buch, Robert-Roessle-Str. 10, 13125 Berlin, Germany.
Corresponding author email address: nazare@fmp-berlin.de

[c]In silico Toxicology and Structural Bioinformatics, Institute of Physiology, Charité Universitätsmedizin Berlin, Virchowweg 6, 10117 Berlin, Germany.

[d]Oslo University Hospital, P.O. Box 4950, Nydalen, Oslo 0424, Norway

[e]Hybrid Technology Hub - Centre of Excellence, Institute of Basic Medical Sciences, University of Oslo, 0317 Oslo, Norway

[f]Symeres Netherlands B.V., Kerkenbos 1013, 6546 BB Nijmegen, the Netherlands

[g]Data Driven Drug Design, Faculty of Mathematics and Computer Sciences, Saarland University, 66123 Saarbrücken, Germany

Corresponding author email address: volkamer@cs.uni-saarland.de

[#]These authors contributed equally

Table of Contents

Deconstruction of an adenosine site binder S3 .... S3
Experimental procedures for the synthesis of the fragments of the adenosine site binder S3 .... S5
Computational and experimental evaluation of molecular properties in linker replacement studies S18
Crystallography S21
WNT/β-catenin signaling reporter assay S22
Recore S22
Molecular docking S23
MD simulation S23
HPLC chromatograms S24
References S26

Deconstruction of an adenosine site binder S3

The adenosine-site binding TNKS2 ligand **S3** was designed by a structure-guided hybridization approach based on two-known ligands.[1] Ligand **S3** showed improved pharmacokinetic properties in mice and dogs and efficacy in a tumor xenograft model. In this study, deconstruction of the hybrid ligand **S3** was demonstrated to analyze the contribution of small fragments of TNKS2 ligand **S3**. Unlike dual-site ligands **1** and **2**, fragmentation of **S3** had deleterious effects on binding. Deconstruction of **S3** from different directions gave 9 small fragments, which were tested against TNKS2. Their binding affinities, Gibbs free-energies, and ligand efficacies are summarized in Table **S1**.

Table S1. Binding parameters of fragments of TNKS2 ligand **S3**[a]

	Structure	K_i	ΔG	LE		Structure	K_i	ΔG	LE
**S3**	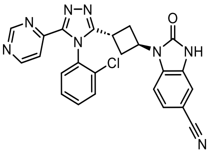	0.006	−46.9	0.33	**S6h**	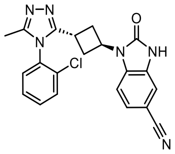	0.26	-37.6	0.31
**S6a**	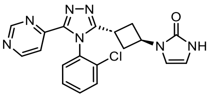	0.1	-40.0	0.35	**S6i**	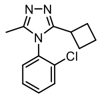	n.a.	-	-
**S6b**	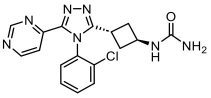	1.1	-34.1	0.32	**S6j**	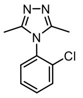	n.a.	-	-
**S6c**	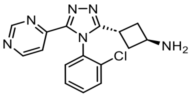	61	-24.1	0.25	**S6k**	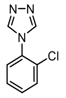	594	-18.4	0.37
**S6d**	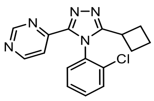	31	-25.7	0.28	**S6l**	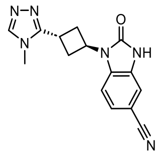	68	-23.7	0.26
**S6e**	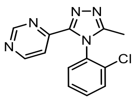	n.a.	-	-	**S6m**	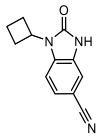	20	-26.7	0.40
**S6f**	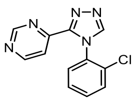	n.a.	-	-	**S6n**	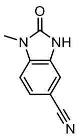	400	-19.4	0.36
**S6g**	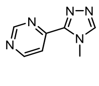	n.a.	-	-	**S6o**	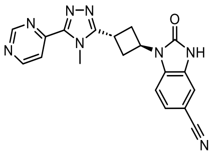	48	-24.6	0.21

[a] K_i values are given in μM. ΔG values are given in kJ/mol, they are calculated at 298 K. Ligand efficiencies (LE) are given in kcal/(mol × number of non-H atoms). [b] n.a. inactive fragments. [c] Estimated ΔG at 298 K for very weak binders from assuming a K_i value >10 mM.

The full ligand **S3** has a K_i of 0.006 and the smallest active fragment is **S6m** with a LE of 0.40 that is also the fragment with the highest LE value in this series. Deletion of three carbons at the cyclobutyl ring of **S6m** gave inactive fragment **S6n**. Fragments **S6e–g** are all lacking the cyclobutyl substituted benzimidazolone fragment **S6m** and did not show activity against TNKS2. Addition of a cyclobutyl ring to the inactive fragment **S6f** showed activity (**S6d** with a LE of 0.30). This can be explained by a possible conformational change within fragments where a cyclobutyl and pyrimidine units forced the 90°rotation of o-chlorophenyl substituent due to the steric hindrance. Furthermore, elongation of the fragment **S6d** to **S6a** increases the LE except the primary amine **6c**. Upon replacement of aryl or heteroaryl groups of ligand **S3** by a single methyl, fragment **S6h** showed higher activity than fragment **6o**, indicating *o*-chlorophenyl is more essential than pyrimidine group to maintain high levels of activity.

Next, ΔG contribution of connecting two fragments were calculated (Table **S2**). Linking fragment **S6d** (ΔG = −25.7 kJ/mol) to fragment **S6n** (ΔG = −19.4 kJ/mol) by removing a methyl group constructs the final ligand **S3** (ΔG = −46.9 kJ/mol). Therefore, ΔG contribution of this operation was found −1.8 kJ/mol. Similar improvement in ΔG was found when connecting fragment **S6f** to fragment **S6m** via a single bond.

Table S2. Calculation of free energy of binding ΔG contribution of linker for ligand **3**[a]

Fragment 1		Fragment 2		ΔG_{frag1}	ΔG_{frag2}	Final ligand	ΔG_{final}	ΔG_{link}[b]	Structural change
**S6d**	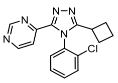	**S6n**	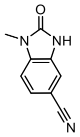	−25.7	−19.4	**S3**	−46.9	-1.8	remove methyl
**S6f**	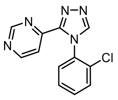	**S6m**	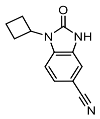	-	-	**S3**	-	-	add single bond

[a] ΔG values are given in kJ/mol, they are calculated at 298 K. [b] The linker contributions are calculated using the formula: $\Delta G_{link} = \Delta G_{final} - \Delta G_{frag1} - \Delta G_{frag2}$.

Experimental procedures for the synthesis of the fragments of the adenosine site binder S3

General information

Flash Column Chromatography was performed on Reveleris® X2 with BÜCHI Flash Pure Silica Cartridges. Reversed Phase Column Chromatography was performed on Reveleris™ prep MPLC with Phenomenex LUNA C18(3) columns using an acetonitrile/water gradient acidified with 0.1% formic acid. LC/MS spectra were recorded on an Agilent 1260 Bin using a Waters XSelect™ C18 (30x2.1mm, 3.5) columns and MS was recorded on Agilent LC/MSD G6130B. Chiral SFC spectra were recorded on Waters Prep100q SFC System using four standard columns; Chiralpak IC (100x4.6mm, 5µm); Phenomenex Cellulose-1 (100x4.6mm, 5µm); Phenomenex Cellulose-2 (100x4.6mm, 5µm); Phenomenex Amylose-1 (100x4.6mm, 5µm). GC-MS spectra were recorded on Agilent 6890N G1530N using a RXi-5MS column (20 m, ID 180 µm, df 0.18 µm) and MS was recorded on MSD 5973 G2577A. ^{1}H-NMR spectra were recorded on a Bruker Avance I – Ultrashield 400 probe: ^{1}H, ^{13}C, COSY, NOESY, HMBC and HSQC spectra were recorded on a Bruker Avance II – Ultrashield 400 probe: ^{1}H, ^{13}C, ^{19}F, ^{15}N, ^{31}P.

Methods

Method Reveleris MPLC-prep (acid): Instrument type: Reveleris™ prep MPLC; Column: Phenomenex LUNA C18(3) (150x25 mm, 10µ); Flow: 40 mL/min; Column temp: room temperature; Eluent A: 0.1% (v/v) Formic acid in water, Eluent B: 0.1% (v/v) Formic acid in acetonitrile.

Method Reveleris MPLC-prep (base): Instrument type: Reveleris™ prep MPLC; column: Waters XSelect CSH C18 (145x25 mm, 10µ); Flow: 40 mL/min; Column temp: room temperature; Eluent A: 10 mM ammoniumbicarbonate in water pH = 9.0); Eluent B: 99% acetonitrile + 1% 10 mM ammoniumbicarbonate in water.

Method LC/MS (AN_Acid): Apparatus: Agilent 1260 Bin. Pump: G1312B, degasser; autosampler, ColCom, DAD: Agilent G1315D, 220-320 nm, MSD: Agilent LC/MSD G6130B ESI, pos/neg 100-1000, ELSD Alltech 3300 gas flow 1.5 ml/min, gas temp: 40°C; column: Waters XSelect^{TM} C18, 50x2.1mm, 3.5 µm, Temp: 35 ºC, Flow: 0.8 mL/min, Gradient: t_0 = 5% A, $t_{3.5min}$ = 98% A, t_{6min} = 98% A, Posttime: 2 min; Eluent A: 0.1% formic acid in acetonitrile, Eluent B: 0.1% formic acid in water.

Method LC/MS (AN_Base): Apparatus: Agilent 1260 Bin. Pump: G1312B, degasser; autosampler, ColCom, DAD: Agilent G1315C, 220-320 nm, MSD: Agilent LC/MSD G6130B ESI, pos/neg 100-1000; column: Waters XSelect^{TM} CSH C18, 50x2.1mm, 3.5 µm, Temp: 25 ºC, Flow: 0.8 mL/min, Gradient: t_0 = 5% A, $t_{3.5min}$ = 98% A, t_{6min} = 98% A, Posttime: 2 min, Eluent A: acetonitrile, Eluent B: 10mM ammoniumbicarbonate in water (pH=9.5).

Chiral SFC Method (IC Column): SFC instrument modules: Waters Prep100q SFC System, PDA: Waters 2998, Fraction Collector: Waters 2767; Column: Chiralpak IC (250x20mm, 5µm), column temp: 35°C; flow: 100 ml/min; ABPR: 170 bar; Eluent A: CO_2, Eluent B: Methanol + 20mM ammonia ; Gradient t_o = 5% B, t_{5min} = 50% B, t_{6min} 50% B, detection: PDA (210-320 nm).

Chiral SFC Method (Cel-1): SFC instrument modules: Waters Prep100q SFC System, PDA: Waters 2998, Fraction Collector: Waters 2767; Column: Phenomenex Lux Cellulose-1 (250x20mm, 5µm), column temp:

35°C; flow: 100 ml/min; ABPR: 170 bar; Eluent A: CO_2, Eluent B: Methanol + 20mM ammonia; Gradient t_o = 5% B, t_{5min} = 50% B, t_{6min} 50% B, detection: PDA (210-320 nm).
Chiral SFC Method (Cel-2): SFC instrument modules: Waters Prep100q SFC System, PDA: Waters 2998, Fraction Collector: Waters 2767; Column: Phenomenex Lux Cellulose-2 (250x20mm, 5µm), column temp: 35°C; flow: 100 ml/min; ABPR: 170 bar; Eluent A: CO_2, Eluent B: Methanol + 20mM ammonia; Gradient t_o = 5% B, t_{5min} = 50% B, t_{6min} 50% B, detection: PDA (210-320 nm).
Chiral SFC Method (AMY1): SFC instrument modules: Waters Prep100q SFC System, PDA: Waters 2998, Fraction Collector: Waters 2767; Column: Phenomenex Lux Amylose-1 (250x20mm, 5µm), column temp: 35°C; flow: 100 ml/min; ABPR: 170 bar; Eluent A: CO_2, Eluent B: Methanol + 20mM ammonia; Gradient t_o = 5% B, t_{5min} = 50% B, t_{6min} 50% B, detection: PDA (210-320 nm).
Method GC/MS (Method A): Instrument: GC: Agilent 6890N G1530N, FID: Det. temp: 300°C and MS: MSD 5973 G2577A, EI-positive, Det.temp.: 280°C Mass range: 50-550; Column: RXi-5MS 20 m, ID 180 µm, df 0.18 µm; Average velocity: 50 cm/s; Injection vol: 1 µl; Injector temp: 250°C; Split ratio: 20/1; Carrier gas: He; Initial temp: 100°C; Initial time: 1.5 min; Solvent delay: 1.3 min; Rate 75°C/min; Final temp 250°C; Hold time 4.5 min.

Preparation of 1-((1R,3r)-3-(4-((S)-2-chlorophenyl)-5-(pyrimidin-4-yl)-4H-1,2,4-triazol-3-yl) cyclobutyl)urea (S6)

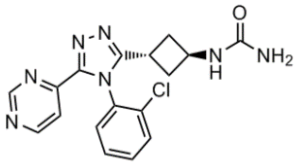

(i) *Tert-butyl ((1R,3r)-3-(4-((S)-2-chlorophenyl)-5-(pyrimidin-4-yl)-4H-1,2,4-triazol-3-yl)cyclobutyl)carbamate* ***(S7)****.* Tert-butyl ((1r,3r)-3-(hydrazinecarbonyl)cyclobutyl)carbamate (1.71 g, 7.48 mmol, 1eq.) was added to a solution of crude methyl N-(2-chlorophenyl)pyrimidine-4-carbimidothioate (2.01 g, 6.84 mmol, 1eq.) in 1-Butanol (40 mL) and was stirred and heated to 140°C for 110 hours. The 1-butanol was evaporated, and the product was purified by Flash Column Chromatography. A 120-gram silica gel cartridge was eluted with a gradient of 0 to 50% dichloromethane/10% methanol in dichloromethane. The fractions of the product were collected, and the dichloromethane was evaporated to provide the yellow-white solid title compound (S**7**) with a yield of 62.6%. ^{1}H NMR (400 MHz, DMSO-*d*$_6$) δ 8.93 (d, J = 5.3 Hz, 1H), 8.86 (d, J = 1.5 Hz, 1H), 8.21 (dd, J = 5.2, 1.4 Hz, 1H), 7.73 – 7.49 (m, 4H), 7.25 (d, J = 7.8 Hz, 1H), 4.20 (q, J = 7.6 Hz, 1H), 3.13 (dt, J = 7.8, 5.1 Hz, 1H), 2.63 (m, 1H), 2.55 – 2.46 (m, 8H, 1H coincides with DMSO signal), 2.13 (dq, J = 7.8, 5.2 Hz, 2H), 1.36 (s, 9H) ppm.
(ii) *(1R,3r)-3-(4-((S)-2-chlorophenyl)-5-(pyrimidin-4-yl)-4H-1,2,4-triazol-3-yl)cyclobutan-1-amine* ***(S6c)*** Hydrogen chloride, 5 to 6N solution in 2-propanol (9.18 mL, 45.9 mmol, 10eq.) was added to a solution of tert-butyl ((1R,3r)-3-(4-((S)-2-chlorophenyl)-5-(pyrimidin-4-yl)-4H-1,2,4-triazol-3-yl)cyclobutyl)carbamate (**7**) (2.00 g, 4.59 mmol, 1eq.) in 40 mL methanol and it was stirred for ±20 hours. The solvents were evaporated

and was dissolved in water and basified with 1 N aqueous sodium hydroxide. The solution was extracted three times with dichloromethane, dried over sodium sulphate, filtered off and evaporated to dryness. The solid was stripped twice with dichloromethane. Finally, it was dissolved in dichloromethane, washed two times with brine, dried with sodium sulphate and evaporated to dryness. The product was purified with Flash Column Chromatography. A 40-gram silica gel cartridge was eluted with a gradient of 0 to 100% dichloromethane/10% methanol + ammonia in dichloromethane. The fractions of the product were evaporated. Compound (**S6c**) was obtained as a yellow sticky glass-like product with a yield of 59% (0.894 g, 2.71 mmol). ^{1}H NMR (400 MHz, chloroform-*d*) δ 8.84 – 8.76 (m, 2H), 8.26 (dd, J = 5.2, 1.4 Hz, 1H), 7.56 – 7.38 (m, 3H), 7.31 – 7.24 (m, 2H), 3.91 (m, 1H), 3.49 (s, 1H), 3.27 (m, 1H), 2.87 – 2.73 (m, 2H), 2.06 – 1.88 (m, 2H), 1.37 (s, 3H) ppm.

(iii) *1-((1R,3r)-3-(4-((S)-2-chlorophenyl)-5-(pyrimidin-4-yl)-4H-1,2,4-triazol-3-yl) cyclobutyl)urea (**S6b**).* To a mixture of (1R,3r)-3-(4-((S)-2-chlorophenyl)-5-(pyrimidin-4-yl)-4H-1,2,4-triazol-3-yl)cyclobutan-1-amine (**S6c**) (102.5 mg, 0.314 mmol, 1eq.) in 186 µL water and 0.184 g crushed ice was added HCl (0.298 mL, 0.298 mmol, 0.95eq.) carefully under nitrogen atmosphere. The reaction was stirred for 1 hour at room temperature. Sodium cyanate (26.3 mg, 0.405 mmol, 1.3eq.) was added and the mixture was stirred for 1 hour, water (400 µL) was added to the suspension and acidified with HCl solution. The solid was filtered off and washed with water. Afterwards, the solid was stirred in water (500 µL) for 30 min, filtered and dried. The solid was purified with Reveleris MPLC-prep acid (Gradient: $t_{0\ min}$ = 5% B, $t_{1\ min}$ = 5% B, $t_{16\ min}$ = 40% B, $t_{17\ min}$ = 100% B, $t_{22\ min}$ = 100% B; Detection UV: 220, 254, 280 nm). The fractions of the product were evaporated and dissolved in acetonitrile/water and dichloromethane for freeze drying (±18 hours). Compound **S6b** was obtained as a white solid with a yield of 10.41% (12.2 mg, 0.033 mmol). ^{1}H NMR (400 MHz, DMSO-d_6) δ 8.93 (d, J = 5.3 Hz, 1H), 8.86 (d, J = 1.4 Hz, 1H), 8.21 (dd, J = 5.4, 1.5 Hz, 1H), 7.69 (td, J = 8.0, 1.6 Hz, 2H), 7.56 (m, 2H), 6.32 (d, J = 7.6 Hz, 1H), 5.39 (s, 2H), 4.27 (q, J = 7.6 Hz, 1H), 3.14 (s, 1H), 2.63 (dt, J = 7.9, 3.8 Hz, 1H), 2.45 (d, J = 7.1 Hz, 21H, 1H coincides with DMSO signal), 2.13 – 1.98 (m, 2H) ppm.

Preparation of 1-((1R,3r)-3-(4-((S)-2-chlorophenyl)-5-(pyrimidin-4-yl)-4H-1,2,4-triazol-3-yl)cyclobutyl)-1,3-dihydro-2H-imidazol-2-one (S6a)

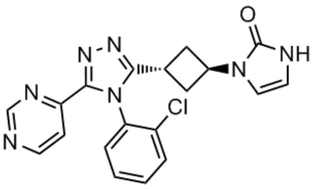

(i) *4-(4-((S)-2-chlorophenyl)-5-((1r,3R)-3-isocyanatocyclobutyl)-4H-1,2,4-triazol-3-yl)pyrimidine (**S8**).* A solution of (1R,3r)-3-(4-((S)-2-chlorophenyl)-5-(pyrimidin-4-yl)-4H-1,2,4-triazol-3-yl) cyclo-butan-1-amine (**S6c**) (100 mg, 0.306 mmol, 1eq.) and triethylamine (0.085 mL, 0.612 mmol, 2eq.) in 3 mL dichloromethane was added dropwise to a solution of bis(trichloromethyl) carbonate (45.9 mg, 0.155 mmol, 0.5eq.) in dichloromethane (1 mL) at 0°C. The mixture was stirred for 1.5 hours at 0°C. The dichloromethane was evaporated, and ethyl acetate was added to the solid. The suspension was filtered off and concentrated to provide the title compound (**S8**) with a yield of 74.1% (80 mg, 0.227 mmol). ^{1}H NMR (400 MHz, chloroform-

d) δ 8.82 (dd, J = 3.4, 1.9 Hz, 2H), 8.27 (dd, J = 5.3, 1.4 Hz, 1H), 7.55 (m, 2H), 7.45 (td, J = 7.6, 1.8 Hz, 1H), 7.30 – 7.23 (m, 6H, 1H coincides with $CDCl_3$ signal), 4.50 – 4.43 (m, 1H), 3.37 – 3.29 (m, 1H), 3.03 – 2.87 (m, 2H), 2.45 – 2.29 (m, 2H) ppm.

(ii) *1-((1R,3r)-3-(4-((S)-2-chlorophenyl)-5-(pyrimidin-4-yl)-4H-1,2,4-triazol-3-yl)cyclobutyl)-3-(2,2-dimethoxyethyl)urea (**S9**).* 4-(4-((S)-2-chlorophenyl)-5-((1r,3R)-3-isocyanatocyclobutyl)-4H-1,2,4-triazol-3-yl)pyrimidine (**S8**) (80 mg, 0.227 mmol, 1eq.) was dissolved in dichloromethane (5 mL). 2,2-dimethoxyethan-1-amine (0.025 mL, 0.227 mmol, 1eq.) was added and the mixture was stirred for 3 hours at room temperature. Dichloromethane was evaporated what resulted in the crude product (**S9**) with a yield of 94% (97.5 mg, 0.213 mmol).

(iii) *1-((1R,3r)-3-(4-((S)-2-chlorophenyl)-5-(pyrimidin-4-yl)-4H-1,2,4-triazol-3-yl)cyclobutyl)-1,3-dihydro-2H-imidazol-2-one (**S6a**).* 1-((1R,3r)-3-(4-((S)-2-chlorophenyl)-5-(pyrimidin-4-yl)-4H-1,2,4-triazol-3-yl)cyclobutyl)-3-(2,2-dimethoxyethyl)urea (**S9**) (97.5 mg, 0.213 mmol) was dissolved in formic acid (0.5 ml, 13.25 mmol) and stirred for 18 hours at room temperature. The formic acid was evaporated, and the product was purified with Reveleris MPLC-prep acid (Gradient: $t_{0\ min}$ = 5% B, $t_{1\ min}$ = 5% B, $t_{16\ min}$ = 50% B, $t_{17\ min}$ = 100% B, $t_{22\ min}$ = 100% B; Detection UV: 210, 254, 275 nm). The fractions were collected and freeze-dried for ±18 hours to afford compound **S6a** with a yield of 35.3% (29.9 mg, 0.075 mmol). ^{1}H NMR (400 MHz, DMSO-*d*$_6$) δ 9.94 (s, 1H), 8.99 – 8.83 (m, 2H), 8.24 (dd, J = 5.3, 1.4 Hz, 1H), 7.72 (m, 2H), 7.58 (m, 2H), 6.72 (t, J = 2.5 Hz, 1H), 6.35 (t, J = 2.7 Hz, 1H), 4.90 (p, J = 8.6 Hz, 1H), 3.17 (dq, J = 9.3, 5.4, 3.8 Hz, 1H), 2.76 – 2.46 (m, 5H) ppm.

Preparation of (1R,3r)-3-(4-((S)-2-chlorophenyl)-5-(pyrimidin-4-yl)-4H-1,2,4-triazol-3-yl)cyclobutan-1-amine (S6c)

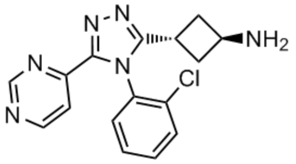

(i) *Tert-butyl ((1R,3r)-3-(4-((S)-2-chlorophenyl)-5-(pyrimidin-4-yl)-4H-1,2,4-triazol-3-yl)cyclobutyl)carbamate (**S7**).* Tert-butyl ((1r,3r)-3-(hydrazinecarbonyl)cyclobutyl)carbamate (**S6**) (1.71 g, 7.48 mmol, 1eq.) was added to a solution of crude methyl N-(2-chlorophenyl)pyrimidine-4-carbimidothioate (**S5**) (2.01 g, 6.84 mmol, 1eq.) in 1-Butanol (40 mL) and was stirred and heated to 140°C for 110 hours. The 1-butanol was evaporated, and the product was purified by Flash Column Chromatography. A 120-gram silica gel cartridge was eluted with a gradient of 0 to 50% dichloromethane/10% methanol in dichloromethane. The fractions of the product were collected, and the dichloromethane was evaporated to provide the yellow-white solid title compound (**S7**) with a yield of 62.6%. ^{1}H NMR (400 MHz, DMSO-*d*$_6$) δ 8.93 (d, J = 5.3 Hz, 1H), 8.86 (d, J = 1.5 Hz, 1H), 8.21 (dd, J = 5.2, 1.4 Hz, 1H), 7.73 – 7.49 (m, 4H), 7.25 (d, J = 7.8 Hz, 1H), 4.20 (q, J = 7.6 Hz, 1H), 3.13 (dt, J = 7.8, 5.1 Hz, 1H), 2.63 (m, 1H), 2.55 – 2.46 (m, 8H, 1H coincides with DMSO signal), 2.13 (dq, J = 7.8, 5.2 Hz, 2H), 1.36 (s, 9H) ppm.

(ii) *(1R,3r)-3-(4-((S)-2-chlorophenyl)-5-(pyrimidin-4-yl)-4H-1,2,4-triazol-3-yl)cyclobutan-1-amine* ***(S6c)*** Hydrogen chloride, 5 to 6N solution in 2-propanol (9.18 mL, 45.9 mmol, 10eq.) was added to a solution of tert-butyl ((1R,3r)-3-(4-((S)-2-chlorophenyl)-5-(pyrimidin-4-yl)-4H-1,2,4-triazol-3-yl)cyclobutyl)carbamate (**7**) (2.00 g, 4.59 mmol, 1eq.) in 40 mL methanol and it was stirred for 20 hours. The solvents were evaporated and was dissolved in water and basified with 1 N aqueous sodium hydroxide. The solution was extracted three times with dichloromethane, dried over sodium sulphate, filtered off and evaporated to dryness. The solid was stripped twice with dichloromethane. Finally, it was dissolved in dichloromethane, washed two times with brine, dried with sodium sulphate and evaporated to dryness. The product was purified with Flash Column Chromatography. A 40-gram silica gel cartridge was eluted with a gradient of 0 to 100% dichloromethane/10% methanol + ammonia in dichloromethane. The fractions of the product were evaporated. Compound (**S6c**) was obtained as a yellow sticky glass-like product with a yield of 59% (0.894 g, 2.71 mmol). ^{1}H NMR (400 MHz, chloroform-*d*) δ 8.84 – 8.76 (m, 2H), 8.26 (dd, J = 5.2, 1.4 Hz, 1H), 7.56 – 7.38 (m, 3H), 7.31 – 7.24 (m, 2H), 3.91 (m, 1H), 3.49 (s, 1H), 3.27 (m, 1H), 2.87 – 2.73 (m, 2H), 2.06 – 1.88 (m, 2H), 1.37 (s, 3H) ppm.

Preparation of 4-(4-(2-chlorophenyl)-5-methyl-4H-1,2,4-triazol-3-yl)pyrimidine (S6e)

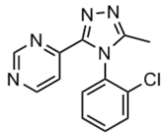

To a solution of methyl N-(2-chlorophenyl)pyrimidine-4-carbimidothioate (197.9 mg, 0.750 mmol, 1eq.) in 1-Butanol (4 ml) was added acetohydrazide (54 mg, 0.729 mmol, 1eq.). The suspension was stirred and heated to 80°C for 20 hours. The solution was heated to 120°C for ±46 hours. The 1-butanol was evaporated, and the product was purified with Reveleris MPLC-prep acid (Gradient: $t_{0\ min}$ = 5% B, $t_{1\ min}$ = 5% B, $t_{2\ min}$ = 10% B, $t_{17\ min}$ = 50% B, $t_{18\ min}$ = 100% B, $t_{23\ min}$ = 100% B; Detection UV: 210, 254, 270 nm). The fractions of the product were collected and freeze-dried for ±20 hours. Compound **S6E** was obtained as a light-yellow solid product with a yield of 35.7% (71.4 mg, 0.260 mmol). ^{1}H NMR (400 MHz, chloroform-*d*) δ 8.85 – 8.78 (m, 2H), 8.24 (dd, J = 5.3, 1.4 Hz, 1H), 7.58 (dd, J = 8.0, 1.6 Hz, 1H), 7.51 (td, J = 7.7, 1.7 Hz, 1H), 7.45 (td, J = 7.6, 1.6 Hz, 1H), 7.32 (dd, J = 7.8, 1.7 Hz, 1H), 2.33 (s, 3H) ppm.

Preparation of methyl N-(2-chlorophenyl)pyrimidine-4-carbimidothioate (S6d)

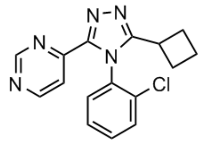

To a solution of methyl N-(2-chlorophenyl)pyrimidine-4-carbimidothioate (127 mg, 0.482 mmol, 1.1eq.) in 1-Butanol (4 ml) followed by cyclobutanecarbohydrazide (**S10b**) (50 mg, 0.438 mmol, 1eq.). The suspension was stirred and heated to 120°C for 100 hours. The 1-butanol was evaporated, and product was dissolved in dichloromethane and purified with Flash Column Chromatography. A 120-gram silica gel cartridge was eluted with a gradient of 0 to 100% dichloromethane/10% methanol in dichloromethane. The fractions were collected,

the dichloromethane was evaporated, and the product was dissolved in acetonitrile/water and freeze dried for 20 hours to afford compound **S6d** as a yellow solid with a yield of 38.2% (54.4 mg, 0.168 mmol). ^{1}H NMR (400 MHz, chloroform-*d*) δ 8.82 – 8.76 (m, 2H), 8.25 (dd, J = 5.3, 1.5 Hz, 1H), 7.57 – 7.39 (m, 3H), 7.28 (dd, J = 7.7, 1.6 Hz, 4H, 1H coincides with $CDCl_3$ signal), 3.27 (p, J = 8.6 Hz, 1H), 2.66 – 2.51 (m, 2H), 2.21 – 1.91 (m, 4H) ppm.

Preparation of 4-(4-methyl-4H-1,2,4-triazol-3-yl)pyrimidine (S6g)

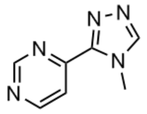

(i) *4-methyl-5-(pyrimidin-4-yl)-4H-1,2,4-triazole-3-thiol (**S12**).* To a solution of pyrimidine-4-carboxylic acid (**1**) (301.9 mg, 2.433 mmol, 1eq.) in N,N-dimethylformamide (dry) (5 mL), 4-Methyl-3-thiosemicarbazide (281.3 mg, 2.67 mmol, 1.1eq.) was added. Di-isopropyl ethylamine (0.763 mL, 4.38 mmol, 1.8eq.) was added drop-wise at room temperature. The mixture was cooled in an ice-bath before adding 1-propanephosphonic cyclic anhydride, 50% w/w in ethyl acetate (2.172 mL, 3.65 mmol, 1.5eq.) (T3P). The reaction was stirred at room temperature for ±18 hours. NaOH (4.6 mL, 9.20 mmol) was added (pH=8). The reaction was diluted with ethyl acetate and the two resulting phases were separated (the upper organic layer was eliminated). The pH was increased to 11 with sodium hydroxide 4M and the mixture was heated to 70°C for 2.5 hours. The solution was cooled with an ice bath and 32% hydrochloric acid was slowly added till pH 5. A precipitate formed, this was filtered under vacuum, washed with water and dried to afford the crude 4-methyl-5-(pyrimidin-4-yl)-4H-1,2,4-triazole-3-thiol (**S12**) as a white solid with a yield of 35.1% (164.9 mg, 0.853 mmol). ^{1}H NMR (400 MHz, DMSO-d_6) δ 14.33 (s, 1H), 9.37 (s, 1H), 9.01 (d, J = 5.2 Hz, 1H), 8.06 (d, J = 5.2 Hz, 1H), 3.89 (s, 3H) ppm.

(ii) *4-(4-methyl-4H-1,2,4-triazol-3-yl)pyrimidine (**S6g**).* To a suspension of 4-methyl-5-(pyrimidin-4-yl)-4H-1,2,4-triazole-3-thiol (**S12**) (164.9 mg, 0.853 mmol, 1eq.) in dichloromethane (30 mL) was added drop-wise at 0°C a solution of hydrogen peroxide (0.087 mL, 0.853 mmol, 1eq.) in acetic acid (1 mL). The mixture was stirred at room temperature for ±18 hours. Additional hydrogen peroxide (0.044 mL, 0.427 mmol, 0.5eq.) in acetic acid (1 mL) was added and the reaction was continued for 26 hours. The dichloromethane was evaporated and was purified with Reveleris MPLC-prep acid (Gradient: $t_{0\ min}$ = 2% B, $t_{1\ min}$ = 2% B, $t_{16\ min}$ = 30% B, $t_{17\ min}$ = 100% B, $t_{22\ min}$ = 100% B; Detection UV: 245, 265, 280 nm). The fractions of the product were collected and freeze dried for ±52 hours. Compound **S6g** was obtained as a white solid with a yield of 41.2% (56.7 mg, 0.352 mmol). ^{1}H NMR (400 MHz, chloroform-*d*) δ 9.29 (d, J = 1.5 Hz, 1H), 8.89 (d, J = 5.3 Hz, 1H), 8.33 (dd, J = 5.3, 1.4 Hz, 1H), 8.25 (s, 1H), 4.19 (s, 3H) ppm.

Preparation of methyl N-(2-chlorophenyl)pyrimidine-4-carbimidothioate (S6f)

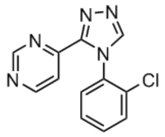

To a solution of methyl N-(2-chlorophenyl)pyrimidine-4-carbimidothioate (**S5**) (166.3 mg, 0.631 mmol, 1eq.) in 1-butanol (5 mL) followed by formohydrazide (41.7 mg, 0.694 mmol, 1.1eq.). The suspension was stirred and heated to 120°C for 28 hours. The temperature was raised to 140°C and the reaction was continued for 26 hours. The 1-butanol was evaporated, and the product was purified with Reveleris MPLC-prep acid (Gradient: $t_{0\ min}$ = 5% B, $t_{1\ min}$ = 5% B, $t_{2\ min}$ = 10% B, $t_{17\ min}$ = 50% B, $t_{18\ min}$ = 100% B, $t_{23\ min}$ = 100% B; Detection UV: 210, 235, 270 nm). The fractions were collected, and the product was freeze-dried over two days (±48 hours) to afford yellow solid with a yield of 39.3% (63.9 mg, 0.248 mmol). ^{1}H NMR (400 MHz, chloroform-*d*) δ 8.91 – 8.83 (m, 2H), 8.34 – 8.26 (m, 2H), 7.59 – 7.37 (m, 4H) ppm.

Preparation of 4-(2-chlorophenyl)-4H-1,2,4-triazole (S6k)

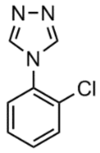

(i) *N-(2-chlorophenyl)-2-formylhydrazine-1-carbothioamide (**S14**).* To a solution of acetohydrazide (74.2 mg, 1.002 mmol, 1.1eq.) in tetrahydrofuran (dry) (20 mL) was added 1-chloro-2-isothiocyanatobenzene (0.115 mL, 0.884 mmol, 1eq.). The mixture was refluxed for 6 hours. The product was collected by filtration and dried with vacuum to afford crude carbothioamide (**S14**) with a yield of 82.0% (167.3 mg, 0.728 mmol).

(ii) *4-(2-chlorophenyl)-4H-1,2,4-triazole-3-thiol (S15).* To a solution of N-(2-chlorophenyl)-2-formylhydrazine-1-carbothioamide (S**14**) (167.3 mg, 0.728 mmol, 1eq.) was added potassium hydroxide (90.1 mg, 1.606 mmol, 2.2eq.) in water (40 mL) and was stirred at 70°C for 2 hours. After cooling, the solution was acidified with 2M HCl solution. The resulting precipitate was collected by filtration and was washed with water. Compound (S**15**) was obtained as a white solid with a yield of 73.6% (113.4 mg, 0.536 mmol).

(iii) *4-(2-chlorophenyl)-4H-1,2,4-triazole (S**6k**).* To a suspension of 4-(2-chlorophenyl)-4H-1,2,4-triazole-3-thiol (**15**) (113.4 mg, 0.536 mmol, 1eq.) in Dichloromethane (30 ml) was added drop-wise at 0°C a solution of hydrogen peroxide (0.103 ml, 1.071 mmol, 2eq.) in Acetic Acid (10 ml). The mixture was stirred at room temperature for 18 hours. The solvents were evaporated, and the product was purified with Reveleris MPLC-prep acid (Gradient: $t_{0\ min}$ = 2% B, $t_{1\ min}$ = 2% B, $t_{16\ min}$ = 30% B, $t_{17\ min}$ = 100% B, $t_{22\ min}$ = 100% B; Detection UV: 210, 220, 230 nm). The fractions were collected, and the product was freeze-dried for 52 hours to afford compound (**S6k**) as a white sticky solid with a yield of 46.3% (45.5 mg, 0.248 mmol). ^{1}H NMR (400 MHz, DMSO-d_6) δ 8.87 (s, 2H), 7.76 (dd, J = 7.5, 2.0 Hz, 1H), 7.66 (dd, J = 7.2, 2.3 Hz, 1H), 7.58 (pd, J = 7.5, 1.9 Hz, 2H) ppm.

Preparation of 1-((1r,3r)-3-(4-methyl-5-(pyrimidin-4-yl)-4H-1,2,4-triazol-3-yl)cyclobutyl)-2-oxo-2,3-dihydro-1H-benzo[d]imidazole-5-carbonitrile (S6o)

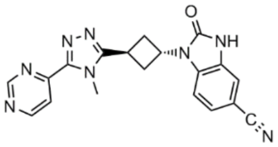

(i) *Pyrimidine-4-carbonyl chloride (**S21**).* A 100 mL round-bottomed flask was charged with pyrimidine-4-carboxylic acid (**1**) (1.0168 g, 8.19 mmol, 1eq.) in dichloromethane (50 ml) followed by oxalyl dichloride (0.774 ml, 9.01 mmol, 1.1 eq.) and N,N-dimethylformamide (dry) (5.0 ml) under nitrogen atmosphere at room temperature. The mixture was stirred at room temperature for 3 hours under nitrogen atmosphere. For samples was methanol added to quench the product. Sample showed 98% conversion of ester product on acid LC-MS and complete conversion on the base LC-MS. It was assumed the reaction had complete conversion and the dichloromethane was evaporated to afford the crude product pyrimidine-4-carbonyl chloride (**S21**) as a dark purple slurry with a yield of 100% (1.168 g, 8.19 mmol).

(ii) *N-methylpyrimidine-4-carboxamide (**S22**).* A solution of methylamine, 2M in THF (4.92 ml, 9.83 mmol, 1.2eq.) in dichloromethane (30 ml) was treated with triethylamine (1.253 ml, 9.01 mmol, 1.1 eq.) and pyrimidine-4-carbonyl chloride (1.168 g, 8.19 mmol, 1eq.). The mixture was stirred for 54 hours. The suspension was washed with water and extracted three times with dichloromethane. The combined extracts were dried over sodium sulphate and the crude compound N-methylpyrimidine-4-carboxamide (**S22**) was obtained with a yield of 79% (884.2 mg, 6.45 mmol).

(iii) *N-methylpyrimidine-4-carbothioamide (**S23**).* A 100 mL flask containing N-methylpyrimidine-4-carboxamide (884.2 mg, 6.45 mmol, 1eq.) was charged with Lawesson's reagent (2710 mg, 6.70 mmol, 1eq.), the flask was placed under ambient atmosphere and toluene (dry) (40 mL) was added. The suspension was heated at reflux for 18 hours. The mixture was cooled down, was filtered off over Celite, toluene was evaporated, and the product was purified with Flash Column Chromatography. A flash column on an 80-gram silica gel cartridge was eluted with a gradient of 0 to 90% DCM in heptane. The fractions of the second batch were collected and the solvents were evaporated to afford *N*-methylpyrimidine-4-carbothioamide (**S23**) as a yellow solid product with a yield of 6.13% (61.2 mg, 0.395 mmol). ^{1}H NMR (400 MHz, chloroform-*d*) δ 10.13 (s, 1H), 9.18 (d, J = 1.5 Hz, 1H), 8.94 (d, J = 5.2 Hz, 1H), 8.57 (dd, J = 5.2, 1.4 Hz, 1H), 3.41 (d, J = 5.2 Hz, 3H) ppm.

(iv) *Methyl (E)-N-methylpyrimidine-4-carbimidothioate (**S24**).* N-methylpyrimidine-4-carbothioamide (61.2 mg, 0.399 mmol, 1eq.) was dissolved in acetone (30 ml), potassium carbonate (77 mg, 0.559 mmol, 1.4eq.) was added followed by a dropwise addition of iodomethane (0.027 ml, 0.439 mmol, 1.1eq.). The suspension was stirred at room temperature over for 56 hours. Additional iodomethane (0.025 ml, 0.399 mmol, 1eq.) was added to the mixture and was stirred for 26 hours. The acetone was evaporated, the residue was dissolved in water and extracted with dichloromethane three times. The orange extracts were dried over sodium sulphate, filtered and evaporated to dryness to afford methyl (E)-N-methylpyrimidine-4-carbimidothioate (**24**) as an orange solution with a yield of 89% (59.5 mg, 0.354 mmol).

*(v) 1-((1r,3r)-3-(4-methyl-5-(pyrimidin-4-yl)-4H-1,2,4-triazol-3-yl)cyclobutyl)-2-oxo-2,3-dihydro-1H-benzo[d]imidazole-5-carbonitrile (**S6o**).* A 10 mL microwave tube was charged with a solution of methyl (E)-N-methylpyrimidine-4-carbimidothioate (59.2 mg, 0.354 mmol, 1eq.) in 1-butanol (4 ml) followed by (1r,3r)-3-(5-cyano-2-oxo-2,3-dihydro-1H-benzo[d]imidazol-1-yl)cyclobutane-1-carbohydrazide (96 mg, 0.354 mmol, 1eq.). The suspension was stirred and heated to 120°C for 18 hours. The solid that was formed was filtered off, washed with water and freeze dried for 18 hours. Compound **S6o** was obtained as a white solid with a yield of 69.9% (92.2 mg, 0.248 mmol). ^{1}H NMR (400 MHz, DMSO-*d*$_6$) δ 11.41 (s, 1H), 9.35 (s, 1H), 8.98 (s, 1H), 8.22 (s, 1H), 7.53 (d, J = 23.2 Hz, 2H), 7.40 (s, 1H), 5.11 (s, 1H), 3.94 (s, 4H), 2.84 (s, 3H) ppm. One signal (1H) not observed most likely coincides with H_2O signal.

Preparation of 1-((1R,3r)-3-(4-(2-chlorophenyl)-5-methyl-4H-1,2,4-triazol -3-yl)cyclobutyl)-2-oxo-2,3-dihydro-1H-benzo[d]imidazole-5-carbonitrile (S6h)

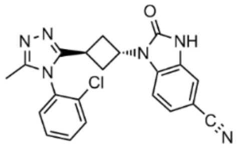

A 10 mL microwave tube was charged with a solution of methyl (E)-N-(2-chlorophenyl)ethanimidothioate (S**31**) (101.1 mg, 0.506 mmol, 1eq.) in 1-butanol (4 ml) followed by (1r,3r)-3-(5-cyano-2-oxo-2,3-dihydro-1H-benzo[d]imidazol-1-yl)cyclobutane-1-carbohydrazide (**S25**) (137 mg, 0.506 mmol, 1eq.). The suspension was stirred and heated to 120°C for 56 hours. 1-butanol was evaporated, and the product was purified with Reveleris MPLC-prep base (Gradient: $t_{0\,min}$ = 5% B, $t_{1\,min}$ = 5% B, $t_{2\,min}$ = 10% B, $t_{17\,min}$ = 50% B, $t_{18\,min}$ = 100% B, $t_{23\,min}$ = 100% B; Detection UV: 220, 265, 285 nm). The fractions were collected and freeze dried for 18 hours to afford compound **S6h** as a white solid with a yield of 52.8% (110.5 mg, 0.267 mmol). ^{1}H NMR (400 MHz, chloroform-*d*) δ 9.32 (s, 1H), 7.64 (dd, J = 7.9, 1.5 Hz, 1H), 7.52 (m, 2H), 7.40 (dd, J = 8.3, 1.5 Hz, 1H), 7.32 (d, J = 1.5 Hz, 1H), 7.26 (dd, J = 7.7, 1.7 Hz, 4H, 1H coincides with $CDCl_3$ signal), 7.18 (d, J = 8.3 Hz, 1H), 5.21 (q, J = 8.8, 8.3 Hz, 1H), 3.43 – 3.29 (m, 3H), 2.87 (m, 2H), 2.27 (s, 3H) ppm.

Preparation of 1-((1r,3r)-3-(4-methyl-4H-1,2,4-triazol-3-yl)cyclobutyl)-2-oxo-2,3-dihydro-1H-benzo[d]imidazole-5-carbonitrile (S6l)

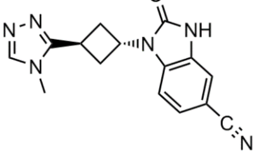

*(i) 2-((1r,3r)-3-(5-cyano-2-oxo-2,3-dihydro-1H-benzo[d]imidazol-1-yl) cyclobutane-1-carbonyl)-N-methylhydrazine-1-carbothioamide (**S26**).* A 50 mL flask was charged with (1r,3r)-3-(5-cyano-2-oxo-2,3-dihydro-1H-benzo[d]imidazol-1-yl)cyclobutane-1-carbohydrazide (**S25**) (103.7 mg, 0.382 mmol, 1eq.) in Ethanol (Abs) (20 ml) followed by isothiocyanatomethane (30.1 mg, 0.412 mmol, 1.1eq.). The suspension was heated under reflux for 2 hours. The mixture was evaporated to dryness to afford crude 2-((1r,3r)-3-(5-cyano-

2-oxo-2,3-dihydro-1H-benzo[d]imidazol-1-yl) cyclobutane-1-carbonyl)-N-methylhydrazine-1-carbothioamide (**S26**) as a solid with a yield of 108% (141.7 mg, 0.411 mmol).

(ii) *1-((1r,3r)-3-(5-mercapto-4-methyl-4H-1,2,4-triazol-3-yl)cyclobutyl)-2-oxo-2,3-dihydro-1H-benzo [d]imidazole-5-carbonitrile (**S27**).* A mixture of 2-((1r,3r)-3-(5-cyano-2-oxo-2,3-dihydro-1H-benzo[d]imidazol-1-yl)cyclobutane-1-carbonyl)-N-methylhydrazine-1-carbothioamide (**S26**) (141.7 mg, 0.411 mmol, 1eq.) and 2M NaOH (8.229 mL, 4.11 mmol, 10eq.) were stirred at room temperature for 21 hours. The solution was acidified to pH 2 with concentrated HCl. The solid that precipitated was filtered off and washed with water to afford 1-((1r,3r)-3-(5-mercapto-4-methyl-4H-1,2,4-triazol-3-yl)cyclobutyl)-2-oxo-2,3-dihydro-1H-benzo [d]imidazole-5-carbonitrile (S**27**) as a white solid with a yield of 57.4% (94.1 mg, 0.411 mmol).

(iii) *1-((1r,3r)-3-(4-methyl-4H-1,2,4-triazol-3-yl)cyclobutyl)-2-oxo-2,3-dihydro-1H-benzo[d]imidazole-5-carbonitrile (**S6l**).* To a suspension of 1-((1r,3r)-3-(5-mercapto-4-methyl-4H-1,2,4-triazol-3-yl)cyclobutyl)-2-oxo-2,3-dihydro-1H-benzo[d]imidazole-5-carbonitrile (**S27**) (94.1 mg, 0.288 mmol, 1eq.) in Dichloromethane (30 ml) was added dropwise at 0 ˚C a solution of hydrogen peroxide (0.065 ml, 0.634 mmol, 2.2eq.) in Acetic Acid (24 ml). The mixture was stirred at room temperature for 18 hours. The dichloromethane was evaporated, and the product was purified with Reveleris MPLC-prep acid (Gradient: $t_{0\ min}$ = 2% B, $t_{1\ min}$ = 2% B, $t_{16\ min}$ = 30% B, $t_{17\ min}$ = 100% B, $t_{22\ min}$ = 100% B; Detection UV: 220, 265, 280 nm). The fractions were collected and freeze dried for 18 hours. Compound (**S6l**) was obtained as a white solid with a yield of 28.2% (24.2 mg, 0.081 mmol). ^{1}H NMR (400 MHz, DMSO-d_6) δ 11.55 (s, 1H), δ 8.43 (s, 1H), 7.55 (d, J = 8.2 Hz, 1H), 7.50 (dd, J = 8.3, 1.6 Hz, 1H), 7.40 (d, J = 1.5 Hz, 1H), 5.09 (p, J = 8.9 Hz, 1H), 3.78 (td, J = 9.8, 4.9 Hz, 1H), 3.54 (s, 3H) ppm. One signal (2H) not observed most likely coincides with H_2O signal.

Preparation of 1-methyl-2-oxo-2,3-dihydro-1H-benzo[d]imidazole-5-carbonitrile (S6n)

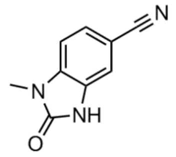

(i) *4-(methylamino)-3-nitrobenzonitrile (**S17**).* 4-fluoro-3-nitrobenzonitrile (**S16**) (1 g, 6.02 mmol, 1eq.) was cautiously added to a solution of methylamine, 2M in THF (3.31 ml, 6.62 mmol, 1.1 eq.). The mixture was stirred at room temperature for 5 hours. The suspension was concentrated and dissolved in dichloromethane. The solution was washed with brine, concentrated and the product was purified with Flash Column Chromatography. A flash column on a 40-gram silica gel cartridge was eluted with a gradient of 0 to 90% ethyl acetate in heptane. The fractions were collected and concentrated to afford 4-(methylamino)-3-nitrobenzonitrile (**S17**) as a yellow solid with a yield of 98% (1.047 g, 5.91 mmol). ^{1}H NMR (400 MHz, chloroform-*d*) δ 8.52 (d, J = 2.0 Hz, 1H), 8.42 (dd, J = 6.7, 2.2 Hz, 1H), 7.64 (dd, J = 7.8, 2.0 Hz, 1H), 6.92 (d, J = 7.8 Hz, 1H), 3.10 (d, J = 5.1 Hz, 3H) ppm.

(ii) *3-amino-4-(methylamino)benzonitrile (**S18**)*. ammonium chloride (158.8 mg, 2.97 mmol, 5.2eq.) was dissolved in water (5.00 ml) and iron powder (170 mg, 3.04 mmol, 5.3eq.) was added. The substrate was dissolved in the organic solvents (water (10.00 ml)/methanol (5.00 ml)/tetrahydrofuran (5.00 ml)). The reaction was heated under an inert atmosphere at 70 °C for 4 hours while vigorously stirring. The organic solvents were removed by evaporation and the solid was stirred with ethyl acetate. The ethyl acetate was decanted, this procedure was repeated once or twice. The combined extracts were dried (brine, sodium sulphate) and evaporated. The product was purified with Flash Column Chromatography. A flash column on a 12-gram silica gel cartridge was eluted with a gradient of 0 to 80% ethyl acetate in heptane. The fractions were collected and concentrated to afford 3-amino-4-(methylamino)benzonitrile as a white-pink solid with a yield of 83% (170 mg, 3.04 mmol). ^{1}H NMR (400 MHz, chloroform-*d*) δ 7.19 (dd, J = 8.2, 1.9 Hz, 1H), 6.93 (d, J = 1.9 Hz, 1H), 6.58 (d, J = 8.2 Hz, 1H), 4.03 (s, 1H), 3.29 (s, 2H), 2.91 (d, J = 4.5 Hz, 3H) ppm.
(iii) *1-methyl-2-oxo-2,3-dihydro-1H-benzo[d]imidazole-5-carbonitrile (**S6n**).* Bis(trichloromethyl) carbonate (134.0 mg, 0.452 mmol, 1eq.) and triethylamine (0.060 mL, 0.433 mmol, 1eq.) were dissolved in tetrahydrofuran (25 mL). A solution of 3-amino-4-(methylamino)benzonitrile (63.8 mg, 0.433 mmol, 1eq.) in tetrahydrofuran (5 mL) was slowly added dropwise at -10 °C. The solution was stirred for 0.5 hours. The pH was adjusted with saturated sodium carbonate solution to pH = 8. Then, it was extracted with ethyl acetate two times, a solid was left behind in the organic layer what appeared to be the product. The product was filtered off and compound **S6n** was obtained as a pink solid with a yield of 51.4% (38.6 mg, 0.223 mmol). ^{1}H NMR (400 MHz, DMSO-d_6) δ 11.28 (s, 1H), 7.50 (dd, J = 8.1, 1.5 Hz, 1H), 7.37 (d, J = 1.5 Hz, 1H), 7.27 (d, J = 8.1 Hz, 1H), 3.32 (s, 4H) ppm.

Preparation of 1-cyclobutyl-2-oxo-2,3-dihydro-1H-benzo[d]imidazole-5-carbonitrile (S6m)

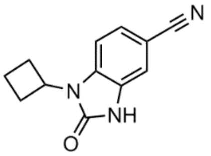

(i) *4-(cyclobutylamino)-3-nitrobenzonitrile* ***(S19).*** 4-fluoro-3-nitrobenzonitrile (105.0 mg, 0.632 mmol, 1eq.) was cautiously added to a solution of cyclobutylamine (331 µl, 0.662 mmol, 1eq.) in dichloromethane (20 ml). The mixture was stirred at room temperature for 5 hours. The suspension was concentrated and dissolved in dichloromethane. The solution was washed with brine, concentrated and purified with Flash Column Chromatography. A flash column on a 12-gram silica gel cartridge was eluted with a gradient of 5 to 50% ethyl acetate in heptane. The fractions were collected and concentrated to afford 4-(cyclobutylamino)-3-nitrobenzonitrile (**S19**) as a yellow solid with a yield of 81% (11.3mg, 0.512 mmol). LC-MS showed a purity of 100% of the end product. No DCM and starting material were found in H-NMR. ^{1}H NMR (400 MHz, chloroform-*d*) δ 8.50 (d, J = 2.0 Hz, 1H), 8.46 (s, 1H), 7.57 (dd, J = 9.0, 2.0 Hz, 1H), 6.77 (d, J = 9.0 Hz, 1H), 4.16 – 4.04 (m, 1H), 2.55 (m, 2H), 2.14 – 2.02 (m, 2H), 2.02 – 1.84 (m, 2H) ppm.

(ii)** 3-amino-4-(cyclobutylamino)benzonitrile **(S20). 4-(cyclobutylamino)-3-nitrobenzonitrile (111.3 mg, 0.512 mmol, 1 eq.) was dissolved in ethyl acetate (20 mL), the mixture was degassed with nitrogen, palladium 10% on activated carbon (32.7 mg, 0.031 mmol, 0.06eq.) was added and the mixture was stirred under a hydrogen atmosphere overnight for 18 hours. The hydrogen was removed using nitrogen, the mixture was filtered over Celite. The ethyl acetate was evaporated to afford 3-amino-4-(cyclobutylamino)benzonitrile **(S20)** as a solid with a yield of 83% (80.8 mg, 0.423 mmol). ^{1}H NMR (400 MHz, chloroform-*d*) δ 7.13 (dd, J = 8.2, 1.9 Hz, 1H), 6.92 (d, J = 1.8 Hz, 1H), 6.48 (d, J = 8.2 Hz, 1H), 4.09 (d, J = 18.0 Hz, 1H), 3.95 (q, J = 6.6 Hz, 1H), 3.29 (s, 2H), 2.52 – 2.41 (m, 2H), 1.96 – 1.79 (m, 4H) ppm.

***(iii)** 1-cyclobutyl-2-oxo-2,3-dihydro-1H-benzo[d]imidazole-5-carbonitrile (**S6m**).* Bis(trichloromethyl) carbonate (**19**) (128 mg, 0.432 mmol, 1eq.) and triethylamine (0.060 mL, 0.432 mmol, 1eq.) were dissolved in tetrahydrofuran (dry) (25 mL). A solution of 3-amino-4-(cyclobutylamino)benzonitrile (80.8 mg, 0.432 mmol, 1eq.) in tetrahydrofuran (dry) (5 mL) was slowly added dropwise at -10 °C. The solution was stirred for 1 hour. The tetrahydrofuran was evaporated, and the residue was dissolved in water and was extracted with ethyl acetate three times. The extracts were evaporated to dryness and the product was purified with Reveleris MPLC-prep acid (Gradient: $t_{0\ min}$ = 2% B, $t_{1\ min}$ = 2% B, $t_{16\ min}$ = 30% B, $t_{17\ min}$ = 100% B, $t_{22\ min}$ = 100% B; Detection UV: 220, 265, 280 nm). The fractions were collected and freeze-dried overnight for 18 hours. Compound **S6m** was obtained as a white solid with a yield of 48.7% (44.8 mg, 0.210 mmol). ^{1}H NMR (400 MHz, chloroform-*d*) δ 9.11 (s, 1H), 7.42 (dd, J = 8.2, 1.6 Hz, 1H), 7.35 – 7.27 (m, 2H), 4.94 – 4.82 (m, 1H), 2.88 (pd, J = 9.8, 2.8 Hz, 2H), 2.44 (m, 2H), 2.06 – 1.85 (m, 2H) ppm.

Preparation of 4-(2-chlorophenyl)-3,5-dimethyl-4H-1,2,4-triazole (S6j)

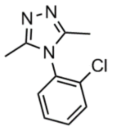

A 10 mL microwave tube was charged with a solution of methyl (E)-N-(2-chlorophenyl)ethanimidothioate (113.3 mg, 0.567 mmol, 1eq.) in 1-butanol (4 ml) followed by acetohydrazide (46.2 mg, 0.624 mmol, 1.1eq.). The suspension was stirred and heated to 120°C for 56 hours. 1-butanol was evaporated, and the product was purified with Reveleris MPLC-prep base (Gradient: $t_{0\ min}$ = 5% B, $t_{1\ min}$ = 5% B, $t_{16\ min}$ = 40% B, $t_{17\ min}$ = 100% B, $t_{22\ min}$ = 100% B; Detection UV: 210, 220, 240 nm). The fractions were collected and freeze-dried overnight for 18 hours to afford compound **S6j** as a white solid with a yield of 52.5% (61.8 mg, 0.298 mmol). ^{1}H NMR (400 MHz, chloroform-*d*) δ 7.64 (dd, J = 7.9, 1.6 Hz, 1H), 7.50 (m, J = 19.3, 7.5, 1.7 Hz, 2H), 7.31 – 7.24 (m, 2H), 2.23 (s, 6H) ppm.

Preparation of 4-(2-chlorophenyl)-3-cyclobutyl-5-methyl-4H-1,2,4-triazole (S6i).

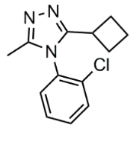

A 10 mL microwave tube was charged with a solution of methyl (E)-N-(2-chlorophenyl)ethanimidothioate (100 mg, 0.501 mmol) in 1-butanol (4 ml) followed by cyclobutanecarbohydrazide (62.9 mg, 0.551 mmol). The suspension was stirred and heated to 120°C for 56 hours. 1-butanol was evaporated, and the product was purified with Reveleris MPLC-prep acid (Gradient: $t_{0\ min}$ = 5% B, $t_{1\ min}$ = 5% B, $t_{2\ min}$ = 10%, B $t_{17\ min}$ = 50% B, $t_{18\ min}$ = 100% B, $t_{23\ min}$ = 100% B; Detection UV: 215, 225, 275 nm). The fractions were collected and freeze-dried overnight for 18 hours. Compound **S6i** was obtained as a white solid with a yield of 56.8% (71.2 mg, 0.285 mmol). ^{1}H NMR (400 MHz, chloroform-*d*) δ 7.61 (dd, J = 7.9, 1.6 Hz, 1H), 7.48 (m, 2H), 7.23 (dd, J = 7.8, 1.7 Hz, 1H), 3.24 – 3.12 (m, 1H), 2.57 – 2.42 (m, 2H), 2.21 (s, 3H), 2.17 – 2.00 (m, 2H), 2.00 – 1.84 (m, 2H) ppm.

Computational and experimental evaluation of molecular properties in linker replacement studies

Table S3: Key characteristics of molecules included in the computational assessment of linker replacement studies. The number of hydrogen bonds with the backbone of the ADE binding pocket as well as the binding affinity score were determined using LigandScout 4.4[2]. The ligand strain was visually estimated by searching for bend aromatic systems and unfavorable bond angles. A molecule was considered stable in MD simulation if the linker did not change its conformation throughout 100 ns of simulation.

Linker design	ID	Structure	number of hydrogen bonds with backbone in ADE pocket	ligand strain	binding affinity score	stable linker in MD simulation
	**1**	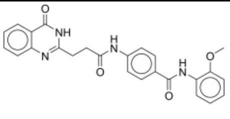	2	no	-48.45	Yes
S01	**6**	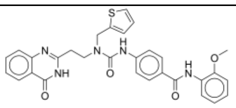	1	no	-58.42	No
S02	**5**	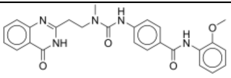	2	no	-42.89	Yes
S03		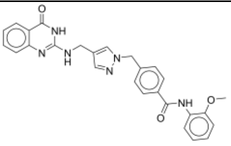	0	yes	-45.09	-
S04		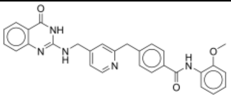	1	yes	-43.32	-
S05		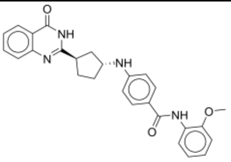	1	no	-48.46	Yes
S06	**10**	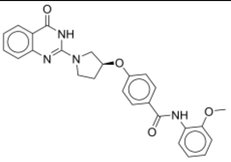	1	no	-46.46	Yes
S07		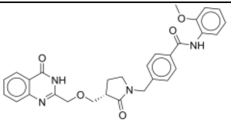	1	yes	-54.65	-
S08		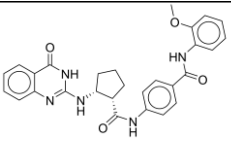	2	no	-44.06	No
S09		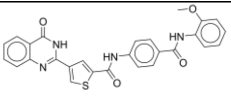	1	yes	-40.45	-

S10		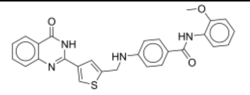	1	yes	-48.68	-
S11		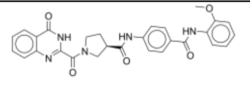	2	yes	-40.63	-
S12		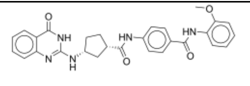	2	no	-43.41	No
S13	**8**	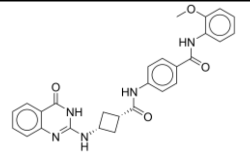	2	no	-47.11	No

Table S4. Binding affinity (p*K*i), clogP and LLE (Ligand-Lipophilicity Efficiency) for all synthesized molecules.

Structure		p*K*i[a]	cLogP[b]	LLE	Structure		p*K*i[a]	cLogP[b]	LLE
**1**	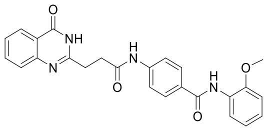	7.28	3.02	4.26	**2**	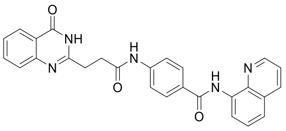	8.42	3.25	5.17
**3a**	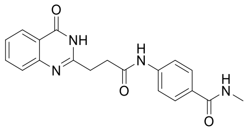	6.04	1.87	4.17	**4a**	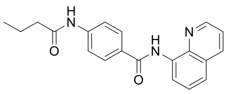	5.92	3.26	2.66
**3b**	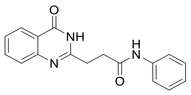	5.21	2.41	2.80	**4b**	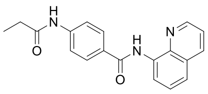	5.38	2.93	2.45
**3c**	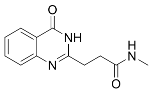	4.64	1.18	3.46	**4c**	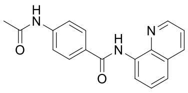	5.38	2.61	2.77
**3d**	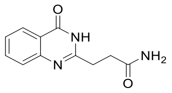	4.57	0.81	3.76	**4d**	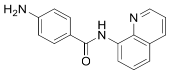	n.a.[c]		
**3e**	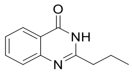	4.34	2.23	2.11	**4e**	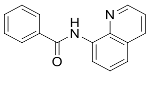	n.a.[c]		

**3f**	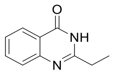	4.39	1.91	2.48	**4f**	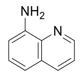	2.96	1.61	1.35
**3g**	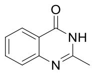	4.43	1.58	2.85	**5**	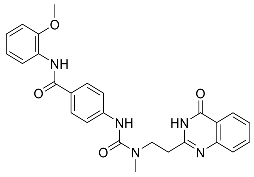	7.06	3.08	3.98
**3h**	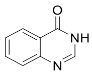	4.05	1.21	2.84	**6**	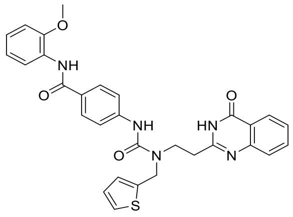	8.07	4.20	3.87
**3i**	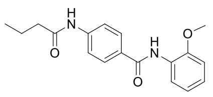	4.57	2.85	1.72	**7**	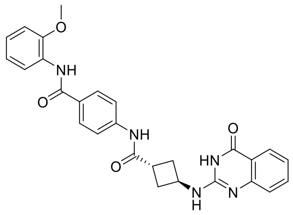	6.06	3.06	3.00
**3j**	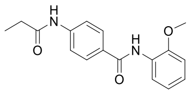	4.13	2.52	1.61	**8**	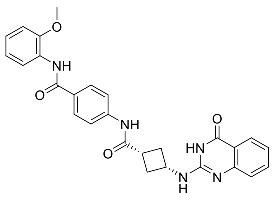	8.00	3.05	4.95
**3k**	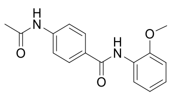	3.84	2.15	1.69	**9**	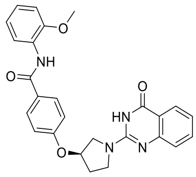	6.72	3.29	3.43
**3l**	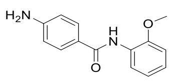	2.92	2.04	0.88	**10**	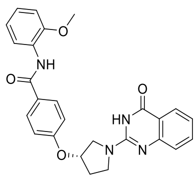	7.47	3.38	4.09
**3m**	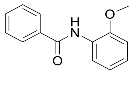	2.68	2.59	0.09					

[a] pK_i for TNKS2 inhibition values. [b] Computational descriptors according to SwissADME.[3] [c] n.a.: inactive fragments.

Crystallography

5.3 mg/ml TNKS2 catalytic domain was mixed with 1:100 chymotrypsin and incubated at 20°C for 2h. The protein was then supplemented with 1 mM of **3k** and mixed with equal volumes of precipitant solution (0.2 M Li_2SO_4, 0.1 M Tris-HCl pH 8.5, 24−26% PEG 3350). The crystallization plate (SwissCI 96-Well 3-Drop plate) was incubated at 4°C. Crystals appeared as clusters of small needles after 1 week. The crystallization droplet was subsequently mixed with 1 mM of **3h** and incubated for 24 hours. For crystallization of TNKS2 in complex with **6**, crystals were grown in the conditions above in the absence of compounds. Three-dimensional crystals of the apo-protein appeared within 1-2 days and were soaked in precipitant solution containing 400 µM **6** for 2 days. The crystals were cryoprotected by soaking in precipitant solution containing additionally 250 mM NaCl and 20% glycerol. Crystals were frozen in liquid nitrogen. Data were collected at ESRF Grenoble on beamline ID30A-1. Diffraction data were processed using the DIALS[4] and XDS[5] packages. Molecular replacement was done using PHASER[6] with the structure of TNKS2 (PDB code: 5NOB) as starting model. Model building and refinement was performed using Coot[7] and Refmac5,[8] respectively. Crystal structure images were prepared using PyMOL Molecular Graphics System (PyMOL, version 1.8.4.0).

Table S5. Data collection and refinement statistics.

**Protein, Inhibitor (PDB id.)**	**TNKS2, 3h & 3k (7OJO)**	**TNKS2, 6 (8B6M)**
**Data collection**		
Beamline	ESRF ID30A-1	ESRF ID30A-1
Wavelength (Å)	0.96546	0.96546
Space group	$P\ 2_12_12_1$	$C222_1$
Crystals	5	1
Unit cell dimensions<br>*a*, *b*, *c* (Å)<br>α, β, γ (°)	 41.645, 76,386, 148.875<br>90, 90, 90	 90.57, 97.37, 119.24<br>90, 90, 90
Resolution range(Å)<br>(Outer cell)	149-2.3 (2.38-2.3)	48.69-1.60 (1.64-1.60)
Total n. of reflections	534876 (40232)	382371 (27362)
N. of unique reflections	21934 (2067)	69406 (51069
Completeness (%)	99.9 (99)	99.7 (99.9)
$\langle I/\sigma(I)\rangle$	5.9 (1.4)	16.0 (1.78)
CC1/2 (%)	98.1 (72.8)	99.9 (64.8)
R_{meas}	0.65 (2.59)	0.057 (1.08)
R_{merge}	0.63 (2.47)	0.052 (0.979)
**Refinement**		
R-work/R-free	0.2382 / 0.2810	0.1849 / 0.2194
**N. of non-hydrogen atoms**		

Protein	3241	3390
Ligands	97	113
Solvent	149	200
**RMSD**		
Bonds (Å)	0.013	0.012
Angles (°)	1.644	1.699
**Average *B* factors (Å^2)**		
Protein	16.8	29.42
Ligands	31.4	40.09
Solvent	13.9	31.74
**Ramachandran plot**		
Favoured (%)	98.73	97.78
Allowed (%)	1.27	1.97
Outliers (%)	0	0.25

*Values within parentheses refers to the highest resolution shell.

WNT/β-catenin Signaling Reporter Assay

Luciferase-based WNT/β-catenin signaling pathway reporter assay was performed as previously described.[9]

Recore

As idea generator for linker replacement or suggestion, the ReCore methodology implemented in SeeSAR [version 11.1.0, BioSolveIT, https://www.biosolveit.de/] was used. Since we observed slightly different orientations of the ligand (4I9I[10]) and the fragments (7OJO, crystal structure from this work) in the tankyrase structures we applied two strategies to identify new linkers: fragment replacement and linkage. (i) For fragment replacement, chain A of PDB structure 4I9I was selected, the default structure preparation was evoked, and the binding site automatically defined based on the ligand 1DY-A (compound **1**). The inspiration mode for core replacements of ReCore was investigated to enumerate compounds replacing the ethyl-amide core of **1** with fragments from the public compound library ZINC (Recore ZINC index 2017).[11] (ii) Similarly for fragment linking, chain B of PDB structure 7OJO was selected, for comparison reason the structure was aligned to 4I9I, default protein preparation was applied, and the binding site automatically determined based on compound **1** from 4I9I. The inspiration mode for linker suggestions was chosen, and the attachment points set at the respective ends of the two co-crystallized fragments **3h** and **3k** (PDB Lig-IDs VGZ-B and WQG-B). For each strategy, the suggested molecules were ranked based on the Hyde Scoring function, and the top 20 molecules were chosen for further investigations. Of the 40 molecules, 11 compounds were selected for synthesis based on further docking experiments and visual inspection (focusing on good scores and important acceptor interaction with Y1060), as well as manually evaluated synthetic feasibility.

Molecular docking

The TNKS2 protein (PDB: 7OJO) of chain A, published with this manuscript, was prepared for molecular docking using the OESpruce 1.3.0.1 and OEChem 3.1.1 toolkits [OpenEye Toolkits 2021.1.1 OpenEye Scientific Software, Santa Fe, NM. http://www.eyesopen.com] and the open-source KinoML library (https://github.com/openkinome/kinoml/), i.e., modeling unresolved side chains and loops, capping of termini with NME and ACE, and protonation at pH 7.4.

Docking of molecules 1 and S01-S13 into the prepared TNKS2 protein structure was performed using the OEDocking toolkit 4.1.0.1 [OpenEye Toolkits 2021.1.1 OpenEye Scientific Software, Santa Fe, NM. http://www.eyesopen.com]. Docking was performed with the hybrid method by transferring the co-crystallized ligand of PDB structure 4I9I[10] into the prepared protein structure published with this manuscript.[12] Conformations of the molecules to dock were generated using the "Pose" option in the Omega toolkit 4.1.1.1 [OpenEye Toolkits 2021.1.1 OpenEye Scientific Software, Santa Fe, NM. http://www.eyesopen.com]. In total, 10 docking poses were retrieved for each docked molecule based on the docking score calculated with the ChemGauss4 scoring function.[13] Subsequently, docking poses were energy minimized in LigandScout 4.4 using the MMFF94 force field and the binding affinity score calculated. [2, 14]

MD simulations

Selected docking poses were submitted to all atomistic unrestrained MD simulations using OpenMM 7.6 [15] together with the Amber14SB and GAFF2.11 force fields.[16, 17] Each protein ligand system was solvated in a cubic box of TIP3P water with 1 nm padding and neutralized with 0.15 M NaCl. The solvated systems were energy minimized to a maximum force cutoff of 10 kJ/mol/nm. Periodic boundary conditions were applied with Particle Mesh Ewald (PME) treatment of long-range interactions with a 1 nm cutoff. Monte Carlo barostat and thermostat were used to maintain pressure and temperature close to 1 bar and 300 K, respectively. A langevin integrator was used with a timestep of 2fs to run unrestrained MD simulations for 105 ns. The first 5 ns were treated as equilibration phase, the final 100 ns were used for analysis. All simulations were run on the high-performance computer from Freie Universität Berlin.[18]

HPLC chromatograms

Recorded via Agilent 1260 series HPLC system, as described in the manuscript

Compound **1** (254 nm)

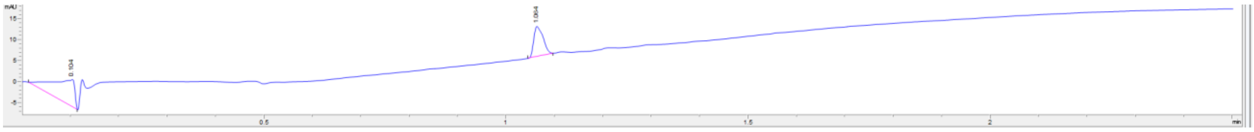

Compound **2** (254 nm)

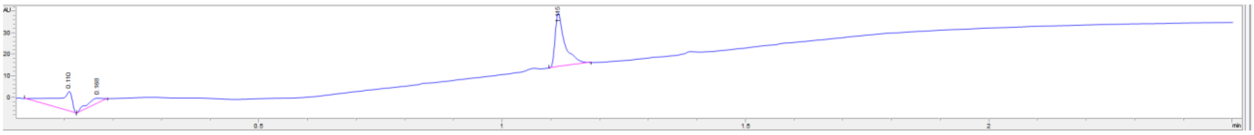

Compound **3a** (254 nm)

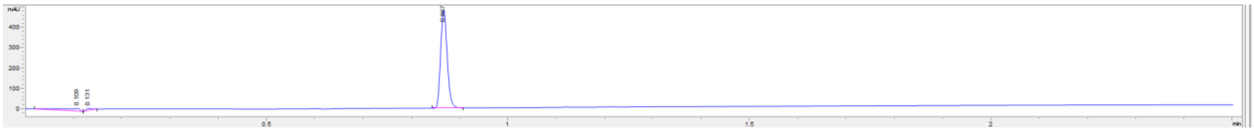

Compound **3b** (254 nm)

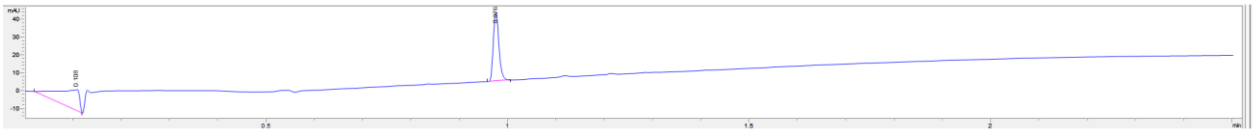

Compound **3d** (254 nm)

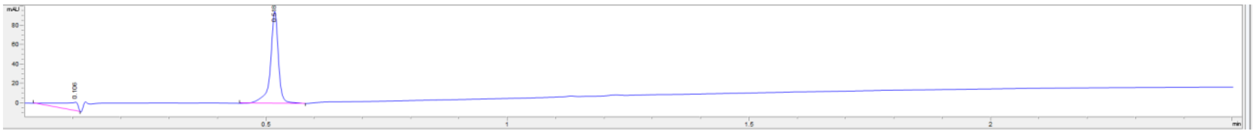

Compound **3i** (254 nm)

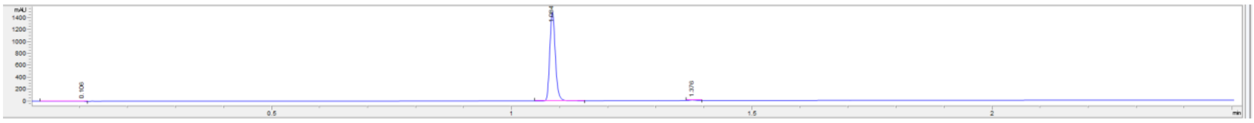

Compound **3k** (254 nm)

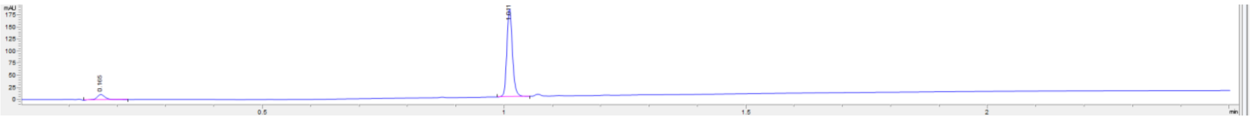

Compound **4a** (254 nm)

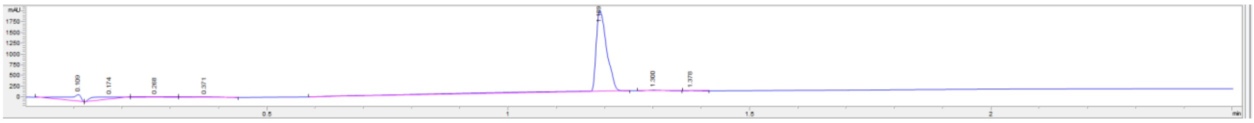

Compound **4c** (254 nm)

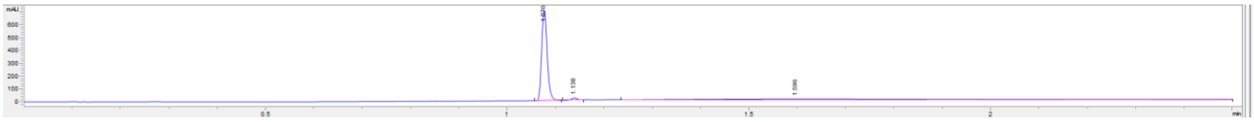

Compound **4d** (254 nm)

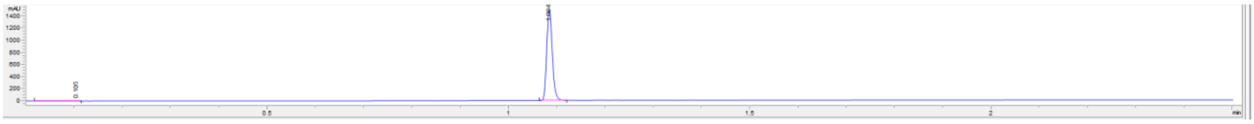

Compound **4e** (254 nm)

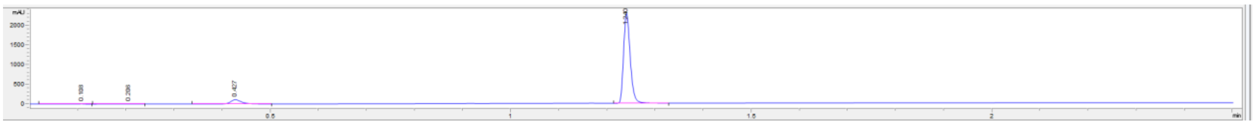

Compound **5** (254 nm)

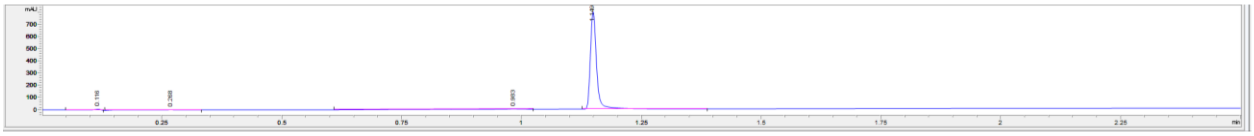

Compound **6** (254 nm)

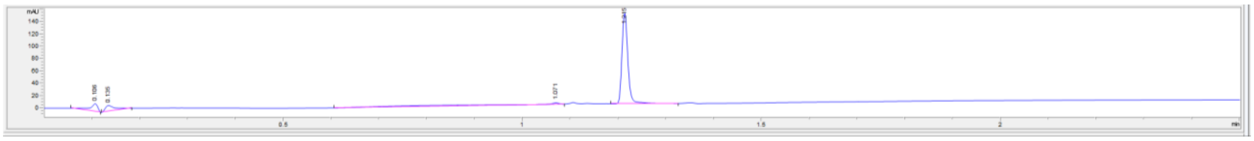

Compound **7** (254 nm)

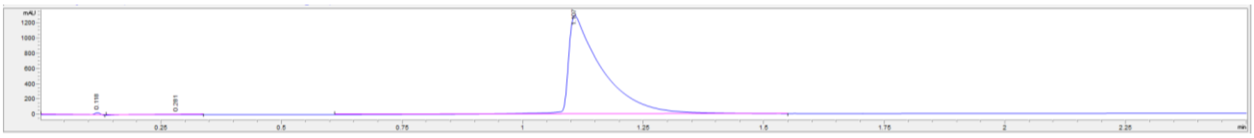

Compound **8** (254 nm)

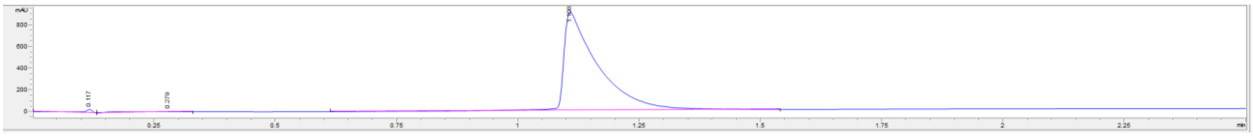

Compound **9** (254 nm)

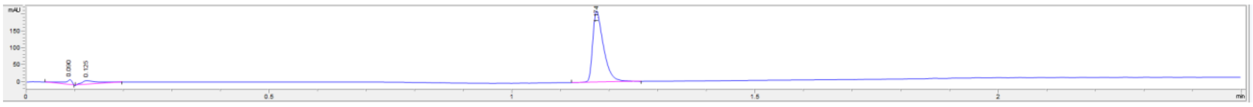

Compound **10** (254 nm)

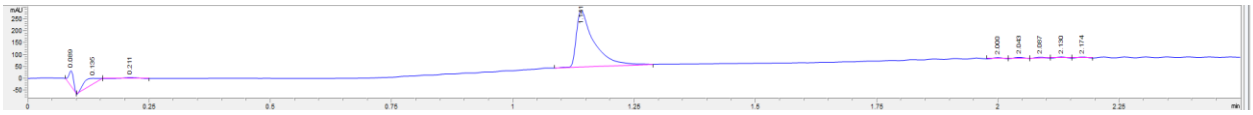

References

(1) Anumala, U. R.; Waaler, J.; Nkizinkiko, Y.; Ignatev, A.; Lazarow, K.; Lindemann, P.; Olsen, P. A.; Murthy, S.; Obaji, E.; Majouga, A. G. Discovery of a novel series of tankyrase inhibitors by a hybridization approach. *Journal of Medicinal Chemistry* **2017**, *60* (24), 10013-10025.
(2) Wolber, G.; Langer, T. LigandScout: 3-D Pharmacophores Derived from Protein-Bound Ligands and Their Use as Virtual Screening Filters. *Journal of Chemical Information and Modeling* **2005**, *45* (1), 160-169.
(3) Daina, A.; Michielin, O.; Zoete, V. SwissADME: a free web tool to evaluate pharmacokinetics, drug-likeness and medicinal chemistry friendliness of small molecules. *Scientific Reports* **2017**, *7* (1), 42717.
(4) Winter, G.; Waterman, D. G.; Parkhurst, J. M.; Brewster, A. S.; Gildea, R. J.; Gerstel, M.; Fuentes-Montero, L.; Vollmar, M.; Michels-Clark, T.; Young, I. D.; Sauter, N. K.; Evans, G. DIALS: implementation and evaluation of a new integration package. *Acta Crystallogr D Struct Biol* **2018**, *74* (Pt 2), 85-97.
(5) Kabsch, W. XDS. *Acta Crystallogr D Biol Crystallogr* **2010**, *66* (Pt 2), 125-132.
(6) McCoy, A. J.; Grosse-Kunstleve, R. W.; Adams, P. D.; Winn, M. D.; Storoni, L. C.; Read, R. J. Phaser crystallographic software. *J Appl Crystallogr* **2007**, *40* (Pt 4), 658-674.
(7) Emsley, P.; Cowtan, K. Coot: model-building tools for molecular graphics. *Acta Crystallogr D Biol Crystallogr* **2004**, *60* (Pt 12 Pt 1), 2126-2132.
(8) Murshudov, G. N.; Skubák, P.; Lebedev, A. A.; Pannu, N. S.; Steiner, R. A.; Nicholls, R. A.; Winn, M. D.; Long, F.; Vagin, A. A. REFMAC5 for the refinement of macromolecular crystal structures. *Acta Crystallogr D Biol Crystallogr* **2011**, *67* (Pt 4), 355-367.
(9) Voronkov, A.; Holsworth, D. D.; Waaler, J.; Wilson, S. R.; Ekblad, B.; Perdreau-Dahl, H.; Dinh, H.; Drewes, G.; Hopf, C.; Morth, J. P.; Krauss, S. Structural Basis and SAR for G007-LK, a Lead Stage 1,2,4-Triazole Based Specific Tankyrase 1/2 Inhibitor. *Journal of Medicinal Chemistry* **2013**, *56* (7), 3012-3023.
(10) Bregman, H.; Gunaydin, H.; Gu, Y.; Schneider, S.; Wilson, C.; DiMauro, E. F.; Huang, X. Discovery of a class of novel tankyrase inhibitors that bind to both the nicotinamide pocket and the induced pocket. *Journal of Medicinal Chemistry* **2013**, *56* (3), 1341-1345.
(11) Sterling, T.; Irwin, J. J. ZINC 15–ligand discovery for everyone. *Journal of chemical information and modeling* **2015**, *55* (11), 2324-2337.
(12) McGann, M. FRED and HYBRID docking performance on standardized datasets. *Journal of Computer-Aided Molecular Design* **2012**, *26* (8), 897-906.
(13) McGann, M. R.; Almond, H. R.; Nicholls, A.; Grant, J. A.; Brown, F. K. Gaussian docking functions. *Biopolymers* **2003**, *68* (1), 76-90.
(14) Halgren, T. A. Merck molecular force field. I. Basis, form, scope, parameterization, and performance of MMFF94. *Journal of Computational Chemistry* **1996**, *17* (5-6), 490-519.
(15) Eastman, P.; Swails, J.; Chodera, J. D.; McGibbon, R. T.; Zhao, Y.; Beauchamp, K. A.; Wang, L.-P.; Simmonett, A. C.; Harrigan, M. P.; Stern, C. D. OpenMM 7: Rapid development of high performance algorithms for molecular dynamics. *PLoS Computational Biology* **2017**, *13* (7), e1005659.
(16) Maier, J. A.; Martinez, C.; Kasavajhala, K.; Wickstrom, L.; Hauser, K. E.; Simmerling, C. ff14SB: Improving the Accuracy of Protein Side Chain and Backbone Parameters from ff99SB. *Journal of Chemical Theory and Computation* **2015**, *11* (8), 3696-3713.
(17) Wang, J.; Wolf, R. M.; Caldwell, J. W.; Kollman, P. A.; Case, D. A. Development and testing of a general amber force field. *Journal of Computational Chemistry* **2004**, *25* (9), 1157-1174.
(18) Bennett, L. M., Bernd; Proppe, Boris. *Curta: A General-purpose High-Performance Computer at ZEDAT, Freie Universität Berlin*. 2020. (accessed 2023/12/18).
